# A Real‐Time Digital Twin for Human Cardiovascular Applications

**DOI:** 10.1002/cnm.70209

**Published:** 2026-09-10

**Authors:** S. C. Snijders, M. C. M. Rutten, J. E. M. Sels, W. A. L. Tonino, W. Huberts

**Affiliations:** ^1^ Department of Biomedical Engineering Eindhoven University of Technology Eindhoven Brabant the Netherlands; ^2^ Department of Medical Physics Laurentius Hospital Roermond Limburg the Netherlands; ^3^ Department of Intensive Care Maastricht UMC+ Maastricht Limburg the Netherlands; ^4^ CARIM Cardiovascular Research Institute Maastricht Maastricht Limburg the Netherlands; ^5^ Department of Cardiology Catharina Hospital Eindhoven Brabant the Netherlands; ^6^ Computational Science Lab, Faculty of Science, Institute for Informatics University of Amsterdam Amsterdam Noord‐Holland the Netherlands

**Keywords:** digital twin, identifiability analysis, real‐time parameter estimation, ROUKF, sensitivity analysis, stability analysis

## Abstract

Digital twin (DT) technology is revolutionizing various industries and has immense potential in healthcare as well. A human DT is a virtual representation of an individual patient, capable of mimicking the current (patho)physiological state and predicting future states, making it ideal for supporting clinical decision‐making. A DT combines a mathematical model with real‐time data; however, its development remains challenging. It must estimate a unique parameter value whenever a measurement becomes available, enabling real‐time tracking of parameter change, while preserving model stability. The DT was designed based on a reduced‐order unscented Kalman filter (ROUKF), using a lumped‐parameter model of the systemic circulation, to estimate left ventricular contractility. This DT was evaluated using both synthetic and in vivo left ventricle pressure data, the latter collected by Johnson et al. Synthetic data were used for local sensitivity, identifiability, and stability analysis, as well as to evaluate the DTs performance. In vivo data were used to evaluate the DTs potential for future clinical application. The DT estimated the true parameter in real‐time, remained robust to measurement noise, and tracked parameter changes independent of initial conditions. The parameter estimate was identifiable despite measurement noise, and the mathematical model proved stable within a physiologically realistic range. Application to in vivo data demonstrated successful tracking of inotropic state changes, with the estimated parameter showing a strong correlation with the conventional contractility proxy (${\left.\frac{\mathrm{dp}}{\mathrm{dt}}\right|}_{\max}$) in multiple patients. Overall, the results demonstrated the DTs potential for real‐time monitoring and personalized medicine, though future work is needed.

## Graphical abstract

A real‐time digital twin was developed using the reduced‐order unscented Kalman filter (ROUKF) to estimate left ventricular contractility from synthetic and in vivo left ventricular pressure measurement data. The results were promising, with identifiable parameter estimates and a stable mathematical model, highlighting the potential for real‐time monitoring and personalized medicine.

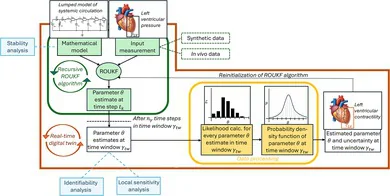

## 1 Introduction

Digital twin (DT) technology is emerging rapidly, driving digital transformation and supporting decision‐making across various industries [1, 2]. This technology is revolutionizing industries owing to its capability to replicate real‐world systems in the digital space [1]. Therefore, DT technology can support a wide array of functions in the industry, such as monitoring, simulation, prediction, and optimization [3]. In healthcare, DT technology offers enormous potential as well, as it can augment the available patient health data, assist in the development of personalized treatment plans, and support data‐driven decision‐making [4, 5, 6].

A human DT is a virtual representation of a physical system, organ, or tissue, capable of mimicking the current (patho)‐physiological state and predicting future states [7]. The mimicking capability enables monitoring of the current state, which is particularly valuable when this state can only be measured invasively or cannot be directly measured at all [8, 9]. Also, disease progression can be simulated based on the prediction of future states, which can be used to support clinical decision‐making [10, 11]. Lastly, combining prediction of future states with the virtual introduction of system changes enables simulation of the patient's response to interventions or treatments, supporting personalization of treatment plans [9]. As a result, human DTs can finally shift the paradigm from decisions based on past experience to predictions driven by a virtual representation of a physiological system [12].

However, developing a DT is challenging in multiple aspects. First of all, the DT must process an in vivo measurement immediately after it becomes available and subsequently provide a parameter estimation before the next measurement arrives, which is referred to as real‐time parameter estimation. Additionally, this DT must capture parameter changes in real‐time, identify unique values for the estimated parameters, and preserve the stability of the mathematical model. The envisioned DT can be created on the basis of data assimilation (DA), a method that merges sparse and possibly noisy measurements with a mathematical model to optimize model predictions based on (recent) patient data [13].

A sequential DA method is based on a time‐stepping approach, in which a model prediction, referred to as the a priori estimate, is updated at each time step based on the discrepancy between the current measurement and model prediction itself, resulting in the a posteriori estimate [13, 14]. This method is able to estimate initial conditions of system states or parameters and update these conditions immediately after a measurement becomes available, making it suitable for real‐time parameter estimation applications such as a DT [15]. In addition, this DA method also provides information about the probability distribution of the estimation, which is considered crucial in clinical decision‐making. One of the most used sequential DA methods is the Kalman filter (KF), a method that provides a recursive estimation based on discrete linear data [13, 16]. However, the evolution of real‐life systems is typically governed by non‐linear equations, which significantly restricts the validity of this linear filter [17].

The unscented Kalman filter (UKF) is an extension of the conventional KF, which can handle non‐linearities with second‐order accuracy [18, 19, 20]. However, extensions like the UKF are known for their computational costs when applied to problems with large amounts of data, making them unsuitable to rapidly evolving complex systems [21]. A computationally more favorable method has been proposed by Moireau et al. [22], in which parameters and states are estimated using distinct methods; the UKF for parameter estimation, and the computationally efficient and inherently stable Luenberger Observer (LO) for state estimation [21, 22, 23]. By combining the UKF and LO following the joint state‐parameter estimation approach, the reduced‐order unscented Kalman filter (ROUKF) can be derived [22]. Given its non‐linear accuracy and computational efficiency, the ROUKF can be considered suitable for designing a real‐time DT.

This study established proof of principle for developing a DT based on a ROUKF for human cardiovascular applications, using synthetic and in vivo data. First, synthetic data were used to conduct three analyses: (I) local sensitivity analysis to identify the sensitivity of the measurement to changes in the parameter of interest, (II) identifiability analysis to evaluate the possibility of identifying a unique parameter value from the measurement, (III) stability analysis to quantify the sensitivity of the system to its initial states. Second, in vivo data were used to demonstrate proof of principle under real‐world conditions. During the recording of these data, a pharmaceutical positive inotropic agent was administered to the patient to provoke different cardiovascular states: rest and pharmacologically induced stress [24]. Demonstrating proof of principle under real‐world conditions requires the DT to track these cardiovascular state changes in real‐time. Finally, for clinical feasibility, it must provide parameter estimates with an acceptable level of confidence while relying on a minimal number of patient measurements.

There are numerous potential cardiovascular applications for this DT, such as veno‐arterial extracorporeal membrane oxygenation (VA‐ECMO), a complex temporary salvage technique used to provide cardiac support to critically ill patients [25]. Detailed mechanistic information on cardiac function is indispensable for decision‐making regarding the timing and feasibility of terminating ECMO support [26, 27]. Consequently, the parameter of interest for this DT was the left ventricular (LV) contractility, a cardiac model parameter representing a scaling factor for the total active stress in the myocardial fiber, serving as an indirect measure for cardiac muscle function [28]. However, ECMO hook‐up was not considered, since this study focused on delivering proof of principle for developing a DT based on a ROUKF.

The mathematical model used in the ROUKF was proposed by Meiburg et al. [29], which was also used to generate the synthetic data in this study. Furthermore, the in vivo data were obtained from Johnson et al. [24]. Meiburg et al. already demonstrated the applicability of the UKF to these specific data, supporting the feasibility of applying the ROUKF to these data. The novelty of the current study lies in the development of a real‐time DT for human cardiovascular applications, combined with the evaluation of parameter identifiability and mathematical model stability, and the use of in vivo data to establish proof of principle under real‐world conditions. The ultimate goal of this DT is clinical applicability, specifically to provide real‐time monitoring of cardiac parameters, to support patient‐specific decision‐making. Eventually, the applicability of a DT extends far beyond real‐time monitoring, as it could play a pivotal role in personalizing medicine and consequently improving patient outcomes.

## 2 Method

The DT was developed based on a ROUKF [22], using the lumped‐parameter model of the systemic circulation of a heart failure patient proposed by Meiburg et al. [29]. The parameter of interest for this DT, with respect to the intended cardiovascular application, was the LV contractility ($c_{\mathrm{LV}}$). Synthetic and in vivo LV pressure ($p_{\mathrm{LV}}$) data were used as input, the former generated by the used mathematical model, the latter obtained from Johnson et al. [24]. Below, the mathematical model, ROUKF, output processing, data and model analysis, measurement data and algorithm re‐initialization, are elaborated in this order.

### 2.1 Mathematical Model

**FIGURE 1:**
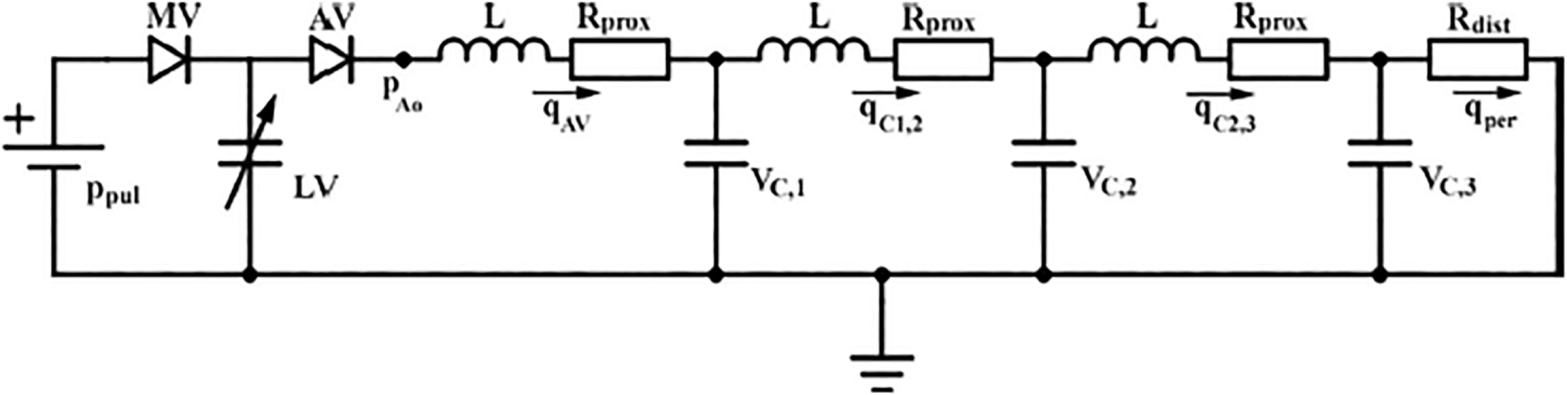
The lumped‐parameter model used to describe the behavior of the cardiovascular system, originating from Meiburg et al. [29].

The model used to describe the behavior of the cardiovascular system originates from Meiburg et al. [29]. This lumped‐parameter model is depicted in Figure 1, in which the LV is connected to the systemic circulation. In this model, the LV behavior is described by the single‐fiber ventricular contraction model proposed by Bovendeerd et al. [28], with a fixed pulmonary pressure as preload. The systemic circulation is modeled as a transmission line of three Windkessel models.

The LV is approximated as an incompressible thick‐walled sphere with muscle fibers oriented in circumferential direction. It is assumed that stretches and stresses are homogeneously distributed in the sphere, enabling global properties (volume and pressure) to be related to local properties of a myocardial fiber (stress and strain). Therefore, the pressure inside the LV ($p_{\mathrm{LV}}$) is related to the stress in fiber direction ($\sigma_{f}$) and radial direction ($\sigma_{r}$), in association with the ratio between LV wall volume ($V_{w}$) and LV cavity volume ($V_{\mathrm{LV}}$), as follows:

(1) $$p_{\mathrm{LV}}=\frac{1}{3}\left(\sigma_{f}-2\sigma_{r}\right)\ln\left(1+\frac{V_{w}}{V_{\mathrm{LV}}}\right).$$

The stress in fiber direction ($\sigma_{f}$) consists of an active ($\sigma_{f,a}$) and a passive component ($\sigma_{f,p}$), while the stress in radial direction ($\sigma_{r}$) is only dependent on passive behavior, which can be stated as:

(2) $$\sigma_{f}=\sigma_{f,a}+\sigma_{f,p}\;\text{and}\;\sigma_{r}=\sigma_{r,p}.$$

Furthermore, the fiber stretch ($\lambda_{f}$) is dependent on wall volume ($V_{w}$), ventricular cavity volume ($V_{\mathrm{LV}}$), and the unloaded cavity volume ($V_{\mathrm{LV},0}$):

(3) $$\lambda_{f}={\left(\frac{V_{\mathrm{LV}}+\frac{1}{3}V_{w}}{V_{\mathrm{LV},0}+\frac{1}{3}V_{w}}\right)}^{\frac{1}{3}}.$$

The myocardial tissue is assumed to be incompressible, resulting in a direct relation between fiber stretch ($\lambda_{f}$) and radial stretch ($\lambda_{r}$), with $\lambda_{f}$ inversely proportional to the square of $\lambda_{r}$. The passive compressive stiffness is assumed to be negligible, resulting in the following definition for passive stress ($\sigma_{i,p}$), in which subscript i denotes the fiber (i=f) or radial (i=r) direction:

(4) $$\sigma_{i,p}=\left\{\begin{array}{ll} \;0, & \lambda_{i}<1,\\ \sigma_{i,0}e^{\left(c_{i}\left(-\lambda_{i}-1\right)-1\right)}, & \lambda_{i}\geq 1. \end{array}\right.$$

The active stress can be calculated as follows:

(5) $$\sigma_{f,a}=c_{\mathrm{LV}}\sigma_{\mathrm{ar}}l_{\mathrm{rel}}\left(l_{s}\right)t_{\mathrm{act}}\left(t_{a}\right)v_{s,\mathrm{rel}}\left(v_{s}\right).$$

The components of this equation; LV contractility ($c_{\mathrm{LV}}$), maximum active stress of healthy tissue ($\sigma_{\mathrm{ar}}$), relative sarcomere length ($l_{\mathrm{rel}}\left(l_{s}\right)$), fiber activation curve ($t_{\mathrm{act}}\left(t_{a}\right)$) and relative sarcomere shortening velocity ($v_{s,\mathrm{rel}}\left(v_{s}\right)$), are expressed in Meiburg et al. [29]. The solution method and the total set of governing equations, collectively representing the mathematical model, are elaborated in Appendix A.

### 2.2 Reduced‐Order Unscented Kalman Filter

The mathematical model is time‐invariant and non‐linear. In this model, the states ${\underset{˜}{x}}_{k}$ $\in {\mathbb{R}}^{n_{s}}$ and parameters ${\underset{˜}{\theta }}_{k}$ $\in {\mathbb{R}}^{n_{p}}$ at time step k are gathered into one state‐parameter space, the augmented state vector ${\underset{˜}{x}}_{\mathrm{aug},k}$ $\in {\mathbb{R}}^{n}$, where $n=n_{s}+n_{p}$. The number of states is denoted by $n_{s}$, the number of parameters by $n_{p}$, and the number of elements in the augmented state vector by n. The evolution of ${\underset{˜}{x}}_{\mathrm{aug},k}$ is governed by the transition function $\underset{˜}{f}\left({\underset{˜}{x}}_{\mathrm{aug},k}\right)$, which is the mathematical model elaborated in Section 2.1 and Appendix A. The measurements are mapped to ${\underset{˜}{x}}_{\mathrm{aug},k}$ using the measurement function $\underset{˜}{h}\left({\underset{˜}{x}}_{k}\right)$, since not all measured states are necessarily equal to state variables. When accounting for model discrepancy ${\underset{˜}{w}}_{k}$ and measurement uncertainty ${\underset{˜}{v}}_{k}$, the time‐invariant non‐linear system reads:

(6) $${\underset{˜}{x}}_{\mathrm{aug},k+1}=\underset{˜}{f}\left({\underset{˜}{x}}_{\mathrm{aug},k}\right)+{\underset{˜}{w}}_{k},\;\text{with}\;{\underset{˜}{x}}_{\mathrm{aug},k}=\left[\begin{array}{c} {\underset{˜}{x}}_{k}\\ {\underset{˜}{\theta }}_{k} \end{array}\right],$$

(7) $${\underset{˜}{y}}_{k}=\underset{˜}{h}\left({\underset{˜}{x}}_{k}\right)+{\underset{˜}{v}}_{k}.$$

Note, in this study, the model discrepancy ${\underset{˜}{w}}_{k}∼N\left(\underset{˜}{0},Q\right)$ and the measurement uncertainty ${\underset{˜}{v}}_{k}∼N\left(\underset{˜}{0},R\right)$ were both assumed to be Gaussian distributed. Also, the model covariance Q and the measurement covariance R were assumed to be time‐invariant, and were chosen arbitrarily in this study. Furthermore, the content of the augmented state vector ${\underset{˜}{x}}_{\mathrm{aug},k}$ and used initialization values are expressed in Appendix A.

Then, the computational efficiency of the ROUKF can be attributed to the application of the joint state‐parameter estimation approach [23], which combines the estimation of ${\underset{˜}{x}}_{k}$ by applying the LO, and the estimation of ${\underset{˜}{\theta }}_{k}$ using the UKF. In the ROUKF, both DA approaches are intertwined and executed simultaneously, which can be projected on ${\underset{˜}{x}}_{\mathrm{aug},k}$ for explanatory purposes as follows:

$${\underset{˜}{x}}_{\mathrm{aug},k}=\left[\begin{array}{c} {\underset{˜}{x}}_{k}\\ {\underset{˜}{\theta }}_{k} \end{array}\right]\begin{array}{c} \to \\ \to \end{array}\;\left.\begin{array}{c} \mathrm{LO}\\ \mathrm{UKF} \end{array}\right\}\text{ROUKF}.$$

The LO is based on a forward nudging approach, a method that adjusts the model output towards the available measurement by introducing a feedback term that captures the discrepancy between the output and the measurement [13, 30]. The UKF retains the recursive approach of the conventional KF, but utilizes the unscented transform (UT) technique to account for non‐linearities. Based on the UT, the distribution of all elements of ${\underset{˜}{x}}_{\mathrm{aug},k}$, described by sigma‐points, are propagated to the next time step using the transition function $\underset{˜}{f}\left({\underset{˜}{x}}_{\mathrm{aug},k}\right)$, which is the mathematical model used [17].

The ROUKF algorithm consists of three recursive steps: (1) the sigma‐point determination, (2) the prediction step, and (3) the analysis step. To elaborate on these three steps: first the sigma‐points are determined using the simplex approach, second the transition function $\underset{˜}{f}\left({\underset{˜}{x}}_{\mathrm{aug},k}\right)$ is used to determine the a priori estimate, ${\underset{˜}{\hat{x}}}_{\mathrm{aug},k+1}^{p}$, and third, this a priori estimate is adapted using the measurement, ${\underset{˜}{y}}_{k+1}$, and results in the a posteriori estimate, ${\underset{˜}{\hat{x}}}_{\mathrm{aug},k+1}^{a}$. A detailed step‐by‐step approach of the ROUKF algorithm is supplied in Appendix B. In the next two subsections, two essential aspects of the reduced‐order method are elaborated: the enhancement of computational efficiency through covariance matrix factorization and the sigma‐point determination.

#### 2.2.1 Covariance Matrix Factorization

The covariances between each pair of elements of the a posteriori estimate ${\underset{˜}{\hat{x}}}_{\mathrm{aug},k+1}^{a}$ are gathered into the a posteriori covariance matrix $P_{k+1}^{a}$. In the ROUKF, the rank of the a posteriori covariance matrix $P_{k+1}^{a}$ $\in {\mathbb{R}}^{n\times n}$ is assumed to be equal to $n_{p}$. This assumption forms the foundation for the reduced‐order method, because it allows rank factorization of $P_{k+1}^{a}$ [22]:

(8) $$P_{k+1}^{a}=L_{k+1}U_{k+1}^{-1}L_{k+1}^{T}.$$

This factorized form, consisting of the Luenberger matrix $L_{k+1}$ $\in {\mathbb{R}}^{n\times n_{p}}$ and the uncertainty matrix $U_{k+1}$ $\in {\mathbb{R}}^{n_{p}\times n_{p}}$, allows computations to be performed on matrices with smaller dimensions, rather than directly on the a posteriori covariance matrix $P_{k+1}^{a}$ itself. This enhances computational efficiency, especially in larger dimensional systems where $n_{s}\gg n_{p}$. The total derivation of this factorized form is outlined in Moireau et al. [22]. However, the reduced rank assumption is only justifiable when the estimation error is allocated to the parameter space ${\underset{˜}{\theta }}_{k}$, which is achieved by concentrating the initial covariance to the parameter space ${\underset{˜}{\theta }}_{k}$. This translates in using the following initial conditions for L and U, with L containing a parameter and a state part, $L^{\theta }$ and $L^{x}$, respectively [22]:

(9) $$L_{k=0}^{\theta }=I,\;L_{k=0}^{x}=O,\;U_{k=0}=I.$$

#### 2.2.2 Simplex Sigma‐Points

Sigma‐points are used to describe the distribution of each element of the augmented state vector ${\underset{˜}{x}}_{\mathrm{aug},k}$, which are determined using the simplex approach. This approach exploits only $n_{p}+1$ sigma‐points to represent a given distribution, arising from the dimensions of the factorized form of the covariance matrix $P_{k+1}^{a}$ (Equation 8) [22]. Application of this simplex approach in the ROUKF involves the simplex sigma‐point matrix S, in which all sigma‐points are located on a regular polyhedron with radius $\sqrt{n_{p}}$, expressed as follows:

(10) $$S^{1}=\left[\begin{array}{cc} -\;\frac{1}{\sqrt{2\varepsilon }} & \frac{1}{\sqrt{2\varepsilon }} \end{array}\right],$$

(11) $$S^{d}=\left[\begin{array}{cccc} & & & 0\\ & S^{d-1} & & \vdots \\ & & & 0\\ \frac{1}{\sqrt{\mathrm{\varepsilon d}\left(d+1\right)}} & \cdots & \frac{1}{\sqrt{\mathrm{\varepsilon d}\left(d+1\right)}} & \frac{-d}{\sqrt{\mathrm{\varepsilon d}\left(d+1\right)}} \end{array}\right],\;2\leq d\leq n_{p}.$$

In this expression, the weight ε is calculated by $\varepsilon =\frac{1}{n_{p}+1}$, with d denoting the dimension index. The weight ε is equal for every element in S, because every sigma‐point is located on the regular polyhedron, implying that the distance to the center of the polyhedron is the same for every sigma‐point. Besides, the mean of every row vector of S is equal to zero, and S has an identity covariance matrix, making it unconstrained and its individual components uncorrelated [22].

### 2.3 Output Processing

The probabilistic nature of the sequentially estimated parameter of interest θ, LV contractility ($c_{\mathrm{LV}}$), was quantified using a probability density function (PDF), which was discretized into $n_{b}$ bins. The PDF was assumed to be log‐normal distributed, since high‐dimensional systems are commonly assumed to be normal distributed [31], and a logarithmic transformation was applied to the parameter of interest to ensure its positive value.

**FIGURE 2:**
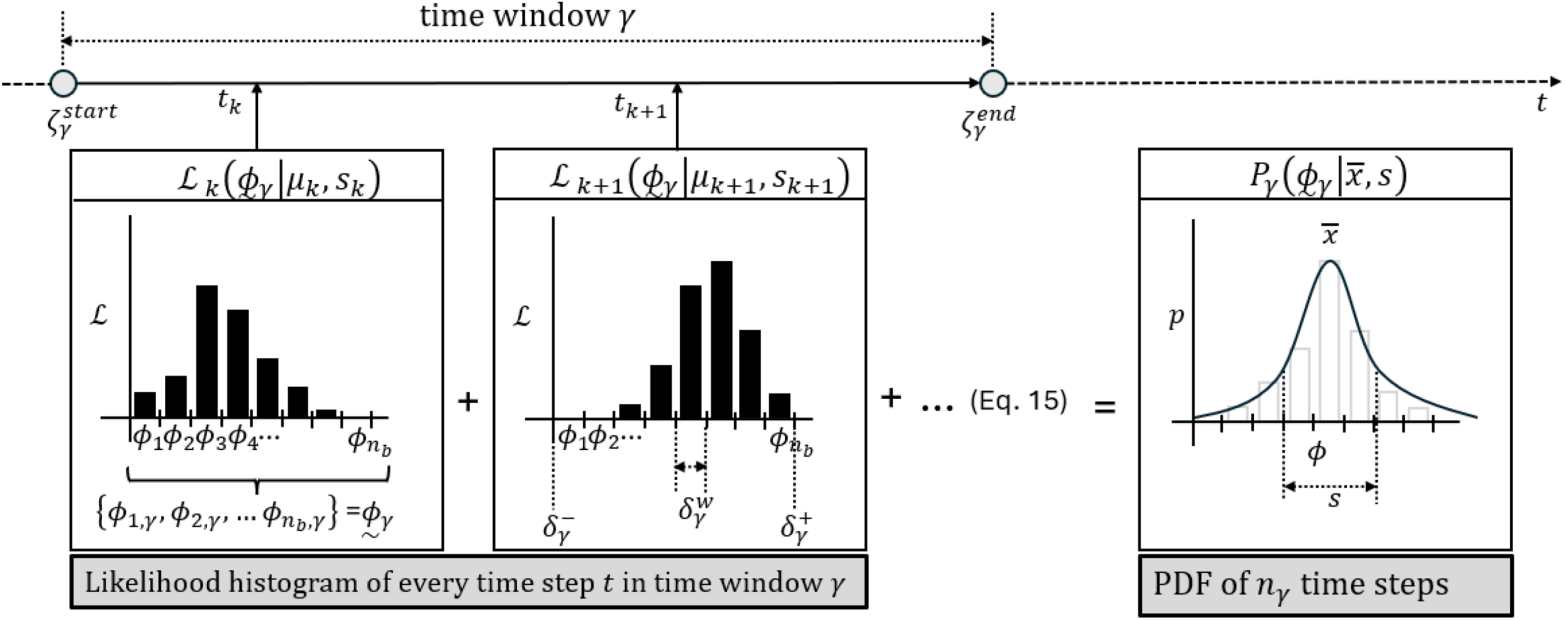
Illustration of the output processing, where the likelihood histograms ${ℒ}_{k}$ of every time step k in time window γ is used to compose the probability density function (PDF) of $n_{\gamma }$ time steps.

The PDF is calculated for every time window γ, ranging from time step $t=\zeta_{\gamma }^{\text{start}}$ to $t=\zeta_{\gamma }^{\mathrm{end}}$, and containing $n_{\gamma }$ time steps. The exact composition of this interval will be elaborated in Section 2.6. Before calculating the PDF of time window γ, a likelihood ℒ histogram is determined for every time step in time interval [$\zeta_{\gamma }^{\text{start}}$, $\zeta_{\gamma }^{\mathrm{end}}$], which is illustrated for time step k and k+1 in the left part of Figure 2.

For every time step k, the likelihood ℒ is calculated for all $n_{b}$ bins, collectively representing the likelihood histogram ${ℒ}_{k}$. Every bin contains a logarithmically transformed parameter value $\phi_{i,\gamma }$, with i indexing the bin and γ indicating the time window. All logarithmically transformed parameter values are gathered in ${\underset{˜}{\phi }}_{\gamma }$ $\in {\mathbb{R}}^{n_{b}}$, which is specific to time window γ and remains constant throughout that time window. This vector is obtained by calculating the lower and upper histogram limits of time window γ, $\delta_{\gamma }^{-}$ and $\delta_{\gamma }^{+}$, and equally dividing this interval into $n_{b}$ bins. The histogram limits, $\delta_{\gamma }^{-}$ and $\delta_{\gamma }^{+}$, are calculated by; (a) identifying the minimum and maximum estimated value of the parameter of interest, ${\hat{\theta }}_{\gamma }^{\min}$ and ${\hat{\theta }}_{\gamma }^{\max}$, within time window γ, (b) calculating the standard deviation of the parameter of interest ${\hat{\sigma }}_{\gamma }$ for time window γ, and eventually (c) determining $\delta_{\gamma }^{-}$ and $\delta_{\gamma }^{+}$ as given below:

(12) $$\delta_{\gamma }^{-}=\left({\hat{\theta }}_{\gamma }^{\min}-3{\hat{\sigma }}_{\gamma }\right)\log_{e}\left(2\right)\;\text{and}\;\delta_{\gamma }^{+}=\left({\hat{\theta }}_{\gamma }^{\max}+3{\hat{\sigma }}_{\gamma }\right)\log_{e}\left(2\right).$$

Next, the number of bins $n_{b}$ is determined using the histogram limits $\delta_{\gamma }^{-}$ and $\delta_{\gamma }^{+}$, together with the bin width $\delta_{\gamma }^{w}$, each of which is specific to the time window γ. In the current study, the bin width $\delta_{\gamma }^{w}$ is calculated using the Freedman–Diaconis rule [32]. Finally, the likelihood histogram ${ℒ}_{k}$ is constructed by evaluating the likelihood of every logarithmically transformed parameter value $\phi_{i,\gamma }$, belonging to bin i and time window γ, given logarithmic transformed parameter estimate $\mu_{k}$ and its corresponding logarithmic standard deviation $s_{k}$, as follows:

(13) $${ℒ}_{k}\left(\phi_{i,\gamma }|\mu_{k},s_{k}\right)=\frac{1}{\phi_{i,\gamma }\sqrt{2\pi }s_{k}}e^{-\frac{1}{2}{\left(\frac{\log_{e}\left(\phi_{i,\gamma }\right)-\mu_{k}}{s_{k}}\right)}^{2}},\;i=1,2,.\ldots n_{b}.$$

The logarithmic transformed parameter estimate $\mu_{k}$, and corresponding logarithmic standard deviation $s_{k}$, are calculated using [33]:

(14) $$\mu_{k}={\hat{\theta }}_{k}\log_{e}\left(2\right)+\log_{e}\left(\theta_{\mathrm{ref}}\right)\;\text{and}\;s_{k}=\sigma_{k}\log_{e}\left(2\right).$$

Here, ${\hat{\theta }}_{k}$ denotes the estimated parameter of interest and $\sigma_{k}$ its corresponding standard deviation, both of which are direct outputs of the ROUKF, while $\theta_{\mathrm{ref}}$ represents the normalization factor. Eventually, after determining the likelihood histogram for all $n_{\gamma }$ time steps in time interval [$\zeta_{\gamma }^{\text{start}}$, $\zeta_{\gamma }^{\mathrm{end}}$], the PDF of time window γ is calculated, which is depicted in the right panel of Figure 2. The PDF of time window γ, denoted as $P_{\gamma }\left({\underset{˜}{\phi }}_{\gamma }|\bar{x},s\right)$, is defined as the weighted product of the likelihood histograms across $n_{\gamma }$ time steps, where the contribution of each step is weighted using a time‐step specific weight $\omega_{k}$ (Equation 17):

(15) $$P_{\gamma }\left({\underset{˜}{\phi }}_{\gamma }|\bar{x},s\right)=\frac{\sum_{k=1}^{n_{\gamma }}\omega_{k}{ℒ}_{k}\left({\underset{˜}{\phi }}_{\gamma }|\mu_{k},s_{k}\right)}{\sum_{k=1}^{n_{\gamma }}\omega_{k}}.$$

For a given time window γ, the estimated parameter of interest θ is defined as the sample mean $\bar{x}$ of the PDF $P_{\gamma }\left({\underset{˜}{\phi }}_{\gamma }|\bar{x},s\right)$, where s denotes the standard deviation. In this study, the estimated θ for time window γ was referred to as estimated $c_{\mathrm{LV}}$, and the confidence level α was set to 0.95.

### 2.4 Data and Model Analysis

#### 2.4.1 Local Sensitivity Analysis

The local sensitivity analysis was used to identify the sensitivity of the measured states to changes in the parameter of interest. More specifically, this method evaluates the effect on the measured state $h_{i,k}$, with measured state i at time step k, when a small disturbance of $\delta h_{i,k}=10^{-4}$ is applied to the estimated parameter of interest ${\hat{\theta }}_{k}$. The local sensitivity $S_{\mathrm{loc},i,k}$ of measured state i at time step k is normalized by measurement‐ and parameter‐specific reference values, $z_{r,h_{i}}$ and $z_{r,\theta }$, as follows:

(16) $$S_{\mathrm{loc},i,k}=\frac{z_{r,\theta }}{z_{r,h_{i}}}\frac{\partial h_{i,k}}{\partial {\hat{\theta }}_{k}},\;1\leq i\leq n_{z}.$$

In this expression, $n_{z}$ represents the number of measured states. Based on the local sensitivity $S_{\mathrm{loc},i,k}$, the contribution of every time step to the PDF (Equation 15) is weighted accordingly, using:

(17) $$w_{k}=\sqrt{\sum_{i=1}^{n_{z}}{\left(S_{\mathrm{loc},i,k}\right)}^{2}}.$$

A small value of the time step specific weight $w_{k}$ indicates that the measured states are not sensitive to a change in parameter value at time step k, implying that this time step contains a negligible amount of valuable information concerning the parameter of interest [34].

#### 2.4.2 Identifiability Analysis

The estimation of the parameter of interest from measurement data represents an inverse problem, for which an identifiability analysis is indispensable. In this study, the identifiability analysis was used to evaluate the possibility to identify an unique value for the parameter of interest based on the measurement data, given a certain model structure [35]. The profile likelihood (PL) method proposed by Raue et al. [36] was applied to perform the identifiability analysis. This method uses a cost function L(θ), defined as follows:

(18) $$L\left(\theta \right)=\sum_{i=1}^{n_{z}}\frac{1}{n_{c}}\frac{1}{n_{\gamma }}\sum_{k=1}^{n_{\gamma }}{\left(\frac{y_{k,i}-{\hat{x}}_{\mathrm{aug},k,i}^{a}}{\sigma \left(e_{i}^{y}\right)}\right)}^{2},\;1\leq i\leq n_{z}.$$

Here, $y_{k,i}$ denotes the measured state and ${\hat{x}}_{\mathrm{aug},k,i}^{a}\left(\theta \right)$ the a posteriori estimate, with i indexing the measured state and k indicating the time step. The agreement between these two quantities is scaled with the standard deviation of the measurement error $\sigma \left(e_{i}^{y}\right)$. Subsequently, the cumulative product of these scaled agreements is averaged over the number of time steps in time window γ, $n_{\gamma }$, and the number of cardiac cycles, $n_{c}$. This process is repeated for every measured state, with the eventual cost function L(θ) containing information of $n_{z}$ measured states. Furthermore, the confidence interval [$\sigma_{\theta }^{-}$, $\sigma_{\theta }^{+}$] of the estimated parameter of interest $\hat{\theta }$ is determined based on confidence level α, following the finite sample method [36]:

(19) $$\left\{\theta |L\left(\theta \right)-L\left(\hat{\theta }\right)<\Delta_{\alpha }\right\},\;\text{with}\;\Delta_{\alpha }=\chi^{2}\left(\alpha ,\mathrm{df}=n_{p}\right).$$

In this study, the threshold $\Delta_{\alpha }$ was defined as the αth quartile of the $\chi_{\mathrm{df}}^{2}$‐distribution. The parameter of interest was considered identifiable if its confidence interval was finite at both bounds.

#### 2.4.3 Stability Analysis

The stability analysis was executed to determine the sensitivity of the mathematical model to its initial states [31]. Model stability is crucial for the convergence of sequential estimates to the actual evolution of the considered system, affecting the performance of the ROUKF [37]. Stability analysis can be conducted using Lyapunov's theorem, whereby Lyapunov's second method is known to be suitable for non‐linear systems governed by ordinary differential equations, making it applicable to the mathematical model used [38]. This method uses a scalar energy‐like function, known as the Lyapunov function $V\left({\underset{˜}{x}}_{\mathrm{aug},k}\right)$, as a substitute for the true energy function, which is often too complicated or even impossible to construct [39]. In this study, the characteristics of the Lyapunov function $V\left({\underset{˜}{x}}_{\mathrm{aug},k}\right)$ were evaluated to assess the stability of the mathematical model. Krasovskii's method was applied to determine the Lyapunov function $V\left({\underset{˜}{x}}_{\mathrm{aug},k}\right)$, which is given by [40]:

(20) $$V\left({\underset{˜}{x}}_{\mathrm{aug},k}\right)=\underset{˜}{f}{\left({\underset{˜}{x}}_{\mathrm{aug},k}\right)}^{T}\underset{˜}{f}\left({\underset{˜}{x}}_{\mathrm{aug},k}\right).$$

The mathematical model $\underset{˜}{f}\left({\underset{˜}{x}}_{\mathrm{aug},k}\right)$ (Section 2.1) contains only first‐order differential equations. Therefore, Krasovskii's method was considered appropriate for determining the Lyapunov function $V\left({\underset{˜}{x}}_{\mathrm{aug},k}\right)$. The mathematical model was considered stable when the time derivative of $V\left({\underset{˜}{x}}_{\mathrm{aug},k}\right)$ was negative definite.

### 2.5 Measurement Data

In this study, a ROUKF was used to estimate a single parameter of interest, LV contractility ($c_{\mathrm{LV}}$), using synthetic and in vivo LV pressure ($p_{\mathrm{LV}}$) data as input, because ventricular pressure is a direct consequence of generated myocardial stress. The synthetic data were generated employing the mathematical model used, which resulted in elimination of model discrepancy, and allowed simulation of specific $c_{\mathrm{LV}}$ values. The in vivo data were obtained from Johnson et al. [24], reflecting a real‐world application of the DT.

#### 2.5.1 Synthetic Data

The $c_{\mathrm{LV}}$ is a model parameter defined as a scaling factor for the total active stress in the myocardial fiber [28]. An appropriate $c_{\mathrm{LV}}$ range for this study was empirically determined by performing simulations using the mathematical model and evaluating cardiac output (CO). According to these simulations, $c_{\mathrm{LV}}$ cannot be lower than approximately 0.3 (CO = 2.5 $\frac{L}{\min}$) to be viable, and can only be higher than approximately 1.5 (CO = 7 $\frac{L}{\min}$) in well‐trained athletes or in patients under inotropic support conditions. Accordingly, the range of $c_{\mathrm{LV}}$ values used in this study was identified at 0.4 to 1.4, with an overview of all simulated scenarios displayed in Table 1.

**TABLE 1:** Simulations of various scenarios to generate the synthetic data that will be used to evaluate the capabilities of the DT.

Test	$c_{\mathrm{LV}}$ (‐)	Noise (%)	Time (s)
1	0.4	—	10
2	0.6	—	10
3	0.8	—	10
4	1.0	—	10
5	1.2	—	10
6	1.4	—	10
7	0.8	10	10
8	0.8	20	10
9	0.8	50	10
10	0.6, 0.8, 1.0	—	30
11	0.6, 0.8, 1.0	20	30
12	1.4, 0.4, 1.2	20	30

In Tests 1–6, different $c_{\mathrm{LV}}$ values were simulated, with the $c_{\mathrm{LV}}$ value remaining constant throughout the test. Based on these tests, the performance of the algorithm with respect to distinct $c_{\mathrm{LV}}$ values was evaluated. In Tests 7 through 9, different levels of noise were added to the data to assess whether the algorithm can cope with noisy measurements. The added noise was Gaussian distributed, with a mean of zero and a standard deviation defined as a percentage of the maximum measured $p_{\mathrm{LV}}$. Table 1 presents the exact percentages for the tests with added noise. Tests 10–12 included a longer simulation time and multiple $c_{\mathrm{LV}}$ values, where a new $c_{\mathrm{LV}}$ value was introduced every 10 s, to evaluate the capability of the algorithm to track $c_{\mathrm{LV}}$ changes over time. In all test scenarios presented in Table 1, synthetic data generation was executed simultaneously with the algorithm on the same computer, acquiring synthetic data at a sampling frequency of 1 kHz. The hardware of the computer used consisted of an Intel Core i7‐7700HQ processor running at 2.8 GHz and 8 GB of RAM. The algorithm was deemed real‐time when the total processing time for each estimate remained smaller than the time interval between data acquisitions.

#### 2.5.2 In Vivo Data

The in vivo data were obtained from Johnson et al. [24]. This study included 16 patients diagnosed with a stenotic aortic valve, with a mean age of 82 years and equal distribution of eight women and eight men. All included subjects provided written informed consent, and the protocol of Johnson's study was approved by the Medical Ethics Committee of the Catharina hospital (Eindhoven, The Netherlands). The dataset consists of 32 invasively acquired LV pressure ($p_{\mathrm{LV}}$) measurement series sampled at 100 Hz, with one measurement series obtained before and one after transcatheter aortic valve implantation (TAVI) for each patient. During every measurement series, changes in inotropic state were elicited by administering dobutamine intravenously following a stepwise approach [24]. Dobutamine is a pharmaceutical positive inotropic agent that increases $c_{\mathrm{LV}}$, leading to an increase in stroke volume, and subsequently an increase in CO [41]. This dataset was considered appropriate to be used as input for the algorithm, since the measured quantity was LV pressure ($p_{\mathrm{LV}}$), and inotropic state changes led to variations in the parameter of interest, LV contractility ($c_{\mathrm{LV}}$). The purpose of applying the algorithm to these in vivo data was not to develop a patient‐specific model, but simply to assess whether the DT is capable of tracking changes in the parameter of interest when using real‐world data. A patient‐specific analysis would require additional patient‐specific data and actual values for the parameter of interest, neither of which were present in the dataset used. Furthermore, to synchronize the cardiac cycle of the mathematical model (Section 2.1) with that of the patient, the onset of ventricular contraction was used as a reference point. This onset, corresponding to the ventricular pressure upstroke, was identified using the $p_{\mathrm{LV}}$ measurement series and defined in this study as a rise above 25 mmHg.

Three different aspects were evaluated using in vivo data to assess proof of principle of the DT under real‐world conditions. First, the ability of the algorithm to follow changes in $c_{\mathrm{LV}}$ over time. It was expected that the $c_{\mathrm{LV}}$ increases during the measurement series due to the stepwise administration of dobutamine. Second, the $p_{\mathrm{LV}}$ measurement data were compared to the a posteriori estimated $p_{\mathrm{LV}}$. The magnitude of discrepancy between the measurement and estimate $p_{\mathrm{LV}}$ served as an indicator for the algorithm's performance. Third, the estimated $c_{\mathrm{LV}}$ was compared with the maximum rate of pressure build up in the LV, LV ${\left.\frac{\mathrm{dp}}{\mathrm{dt}}\right|}_{\max}$, because LV ${\left.\frac{\mathrm{dp}}{\mathrm{dt}}\right|}_{\max}$ is used as a surrogate marker for $c_{\mathrm{LV}}$ in clinical practice [42]. The LV ${\left.\frac{\mathrm{dp}}{\mathrm{dt}}\right|}_{\max}$ was calculated for each cardiac cycle using the measurement data and subsequently averaged over 10 s time windows for comparison with the estimated $c_{\mathrm{LV}}$, which was determined over the same time windows. Both the estimated $c_{\mathrm{LV}}$ and LV ${\left.\frac{\mathrm{dp}}{\mathrm{dt}}\right|}_{\max}$ time series were smoothed using a third‐order Savitzky–Golay filter with a window size of 25 samples [43] to reduce short‐term variability. The Pearson correlation coefficient (r) was used to quantify the linear association between the smoothed $c_{\mathrm{LV}}$ and LV ${\left.\frac{\mathrm{dp}}{\mathrm{dt}}\right|}_{\max}$ time series [44], thereby assessing the degree to which both parameters exhibited similar temporal changes. Based on the Pearson correlation coefficient, measurement series were categorized into two groups: strong correlation (r ≥ 0.7) and moderate‐to‐weak correlation (r < 0.7) [45, 46].

### 2.6 Algorithm Re‐Initialization

The ROUKF algorithm sequentially estimates the parameter of interest using measurement data, in which the Luenberger matrix $L_{k}$ and the uncertainty matrix $U_{k}$ play key roles in terms of parameter behavior. These matrices reflect historic development of the parameter of interest, as they are derived from the sigma‐point matrices, which incorporate information from the previous time steps. After several iterations the parameter of interest converges to its steady‐state value ($\dot{\theta }=0$), which is accompanied by the $L_{k}$ and $U_{k}$ matrices exhibiting small and nearly constant values, leading to a similar behavior of the covariance matrix $P_{k}$. In this steady‐state, the algorithm becomes insensitive to parameter changes, resulting in failure to track parameter changes over time.

To preserve near real‐time precision over time, re‐initialization of $L_{k}$ and $U_{k}$ was proposed. Consequently, the algorithm was reinitialized at the first iteration of every time window, which has a length of $\tau_{1}=10s$, equivalent to $n_{\tau_{1}}$ number of time steps. During re‐initialization, $L_{k=n_{\tau_{1}}}$ and $U_{k=n_{\tau_{1}}}$ were set to their initial conditions (Equation 9), while all other elements of the algorithm remained unaffected. However, re‐initialization of these two matrices may cause transient effects that contain no truthful information about the parameter of interest, which is why the first $\tau_{2}=5s$ of every time window were excluded from further analysis. As a result, time window γ is used for output processing, which contains $n_{\gamma }$ number of time steps, and includes time interval:

(21) $$\left[\zeta_{\gamma }^{\text{start}},\zeta_{\gamma }^{\mathrm{end}}\right]=\left[\tau_{2}+\left(\gamma -1\right)\tau_{1},\tau_{1}+\left(\gamma -1\right)\tau_{1}\right],\;\text{with}\;\gamma =1,2,3..\ldots n_{\mathrm{tw}}$$

This leads to a ‘semi’ real‐time update of the parameter of interest, which is determined for every time window γ, with the total number of time windows $n_{\mathrm{tw}}$ depending on the algorithm's execution time. Lastly, the root‐mean‐square error (RMSE) was used to quantify the discrepancy between the estimates of the parameter of interest ${\hat{\theta }}_{k}$ within time window γ and the final parameter prediction $\bar{x}$.

## 3 Results

### 3.1 Synthetic Data

**FIGURE 3:**
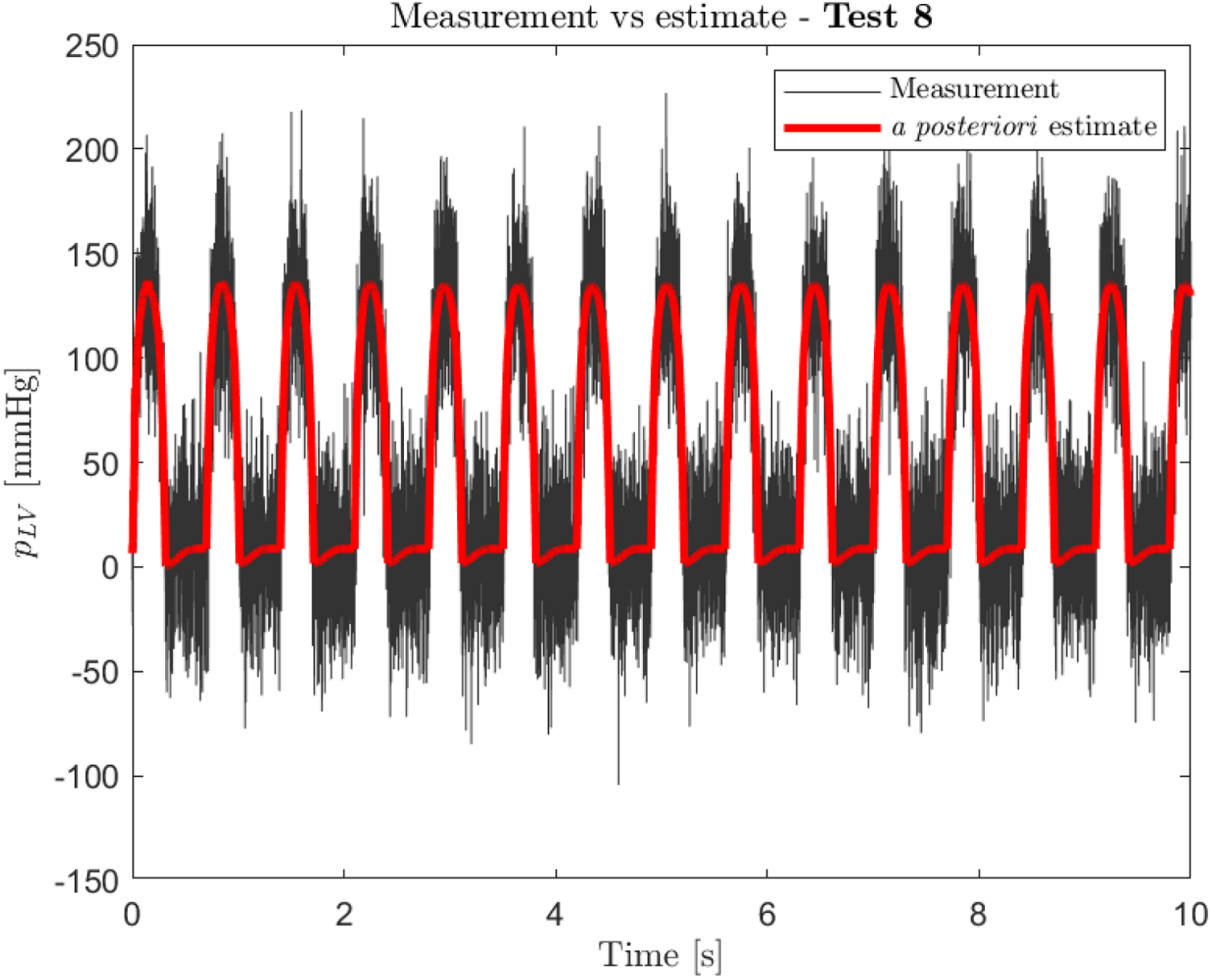
Comparison of the measured $p_{\mathrm{LV}}$ and the a posteriori estimated $p_{\mathrm{LV}}$ of Test 8.

In this section, the results of the application of the ROUKF‐based algorithm to synthetic data were elaborated first, with an overview of all simulated scenarios being presented in Table 1. In all simulated scenarios the synthetic data generation and the algorithm were executed simultaneously on the same computer. In all scenarios, no time lag was observed, implying that the used hardware was able to execute the algorithm in real‐time. The results of the application of the algorithm to Test 1 through 9 are displayed in Table 2. All tests depict a narrow confidence interval (CI $c_{\mathrm{LV}}$), with a range around 0.01, along with a negligible RMSE and difference of $c_{\mathrm{LV}}$ (Diff $c_{\mathrm{LV}}$). Figure 3 illustrates the performance of the algorithm concerning Test 8, a typical noisy measurement example, where the measured $p_{\mathrm{LV}}$ is plotted against the a posteriori estimated $p_{\mathrm{LV}}$. In this figure, the a posteriori estimated $p_{\mathrm{LV}}$ is not visibly affected by the noise‐containing $p_{\mathrm{LV}}$ measurement, which is in line with the consistent algorithm behavior across all tests with noisy measurements, Tests 7 through 9.

**TABLE 2:** Results of the application of the algorithm to the synthetic data of Tests 1 through 9.

Test	True $c_{\mathrm{LV}}$ (‐)	Noise (%)	Estimated $c_{\mathrm{LV}}$ (‐)	CI $c_{\mathrm{LV}}$ (‐)	RMSE	Diff. $c_{\mathrm{LV}}$ (‐)
1	0.4	—	0.400	[0.390, 0.410]	0	0
2	0.6	—	0.600	[0.594, 0.606]	0	0
3	0.8	—	0.801	[0.795, 0.807]	0	0.001
4	1.0	—	1.001	[0.997, 1.005]	0	0.001
5	1.2	—	1.201	[1.197, 1.205]	0	0.001
6	1.4	—	1.401	[1.397, 1.405]	0	0.001
7	0.8	10	0.802	[0.796, 0.808]	0.001	0.002
8	0.8	20	0.798	[0.792, 0.804]	0.001	−0.002
9	0.8	50	0.806	[0.796, 0.816]	0.002	0.006

*Note:* All included tests were simulated for a duration of 10 s. The estimated $c_{\mathrm{LV}}$ was calculated based on the PDF, with the Diff. $c_{\mathrm{LV}}$ reflecting the difference between the estimated and the true $c_{\mathrm{LV}}$. In Tests 7–9, noise was added to the measurement, which was Gaussian distributed with a mean of zero and a test‐specific standard deviation.

**FIGURE 4:**
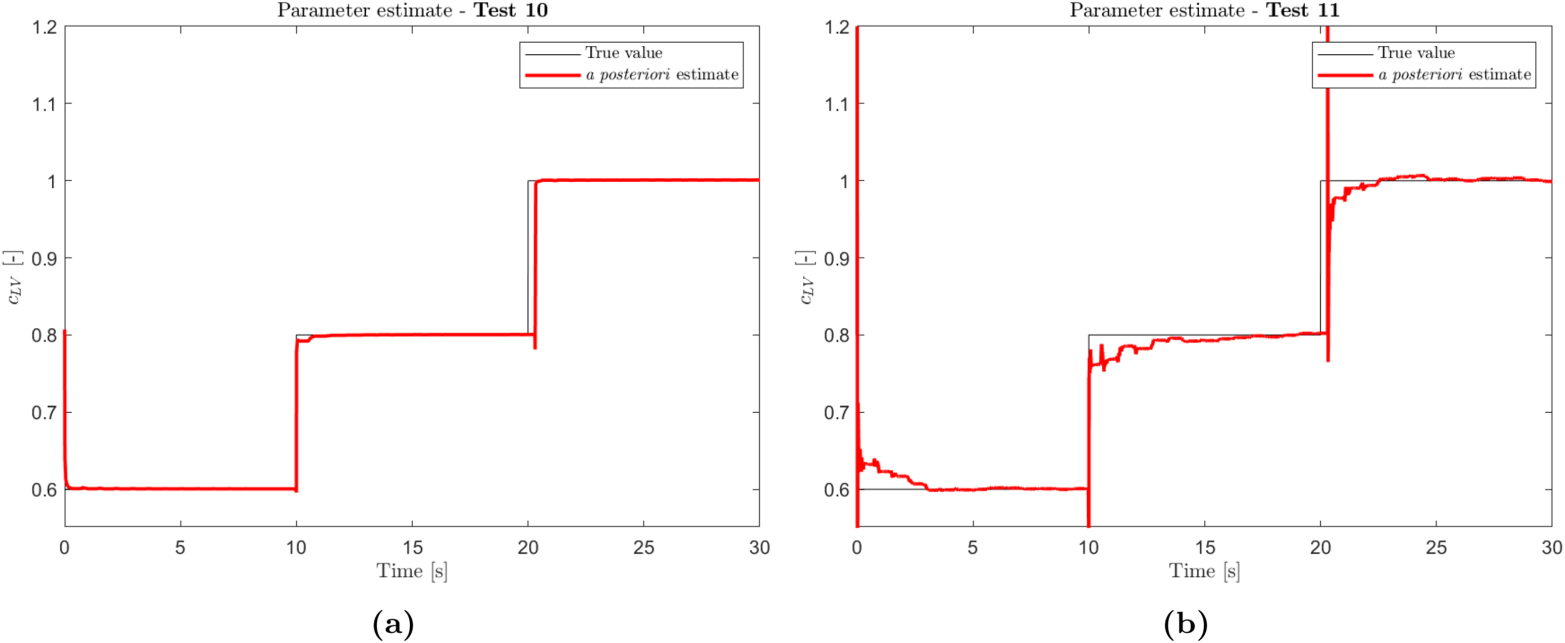
Illustration of the a posteriori estimated $c_{\mathrm{LV}}$ plotted against the true $c_{\mathrm{LV}}$ concerning (a) Test 10 and (b) Test 11 over time.

The results of the application of the algorithm to Tests 10 through 12 are depicted in Table 3, which shows that all three tests contain low RMSE values and minor $c_{\mathrm{LV}}$ differences. These tests were used to evaluate whether the algorithm could handle extreme $c_{\mathrm{LV}}$ changes, despite being physiologically unrealistic scenarios. In all three tests, the algorithm converged to the true $c_{\mathrm{LV}}$ and followed the simulated changes in $c_{\mathrm{LV}}$. Figure 4a,b depict the a posteriori estimated $c_{\mathrm{LV}}$ of Test 10 and 11 respectively, illustrating the algorithm's performance with respect to different noise levels. The re‐initialization of the algorithm introduced transient effects, which were more prominent when measurement noise was present (Test 11), as compared to its absence (Test 10). However, the presence of the transient effects did not influence the a posteriori estimated $c_{\mathrm{LV}}$, as only the latter part of the time window was used for PDF calculation. For the sake of completeness, Test 12 depicted similar transient effects as Test 11, only with different simulated $c_{\mathrm{LV}}$ values.

**TABLE 3:** Results of the application of the algorithm to the synthetic data of Tests 10 through 12.

Test	True $c_{\mathrm{LV}}$ (‐)	Noise (%)	Estimated $c_{\mathrm{LV}}$ (‐)	CI $c_{\mathrm{LV}}$ (‐)	RMSE	Diff. $c_{\mathrm{LV}}$ (‐)
10	0.6	—	0.601	[0.595, 0.607]	0	0.001
	0.8	—	0.800	[0.794, 0.806]	0	0
	1.0	—	1.001	[0.997, 1.005]	0	0.001
11	0.6	20	0.602	[0.592, 0.612]	0.002	0.002
	0.8	20	0.796	[0.790, 0.802]	0.001	−0.004
	1.0	20	1.002	[0.994, 1.010]	0.002	0.002
12	1.4	20	1.403	[1.399, 1.407]	0.002	0.003
	0.4	20	0.398	[0.374, 0.422]	0.004	−0.002
	1.2	20	1.205	[1.199, 1.211]	0.003	0.005

*Note:* All included tests were simulated for a duration of 30 s, with every row representing 10 s of the total simulation time. The estimated $c_{\mathrm{LV}}$ was calculated based on the PDF, with the Diff. $c_{\mathrm{LV}}$ reflecting the difference between the estimated and the true $c_{\mathrm{LV}}$. In Tests 11 and 12, noise was added to the measurement, which was Gaussian distributed with a mean of zero and a test‐specific standard deviation.

### 3.2 Local Sensitivity, Identifiability, and Stability Analysis

**FIGURE 5:**
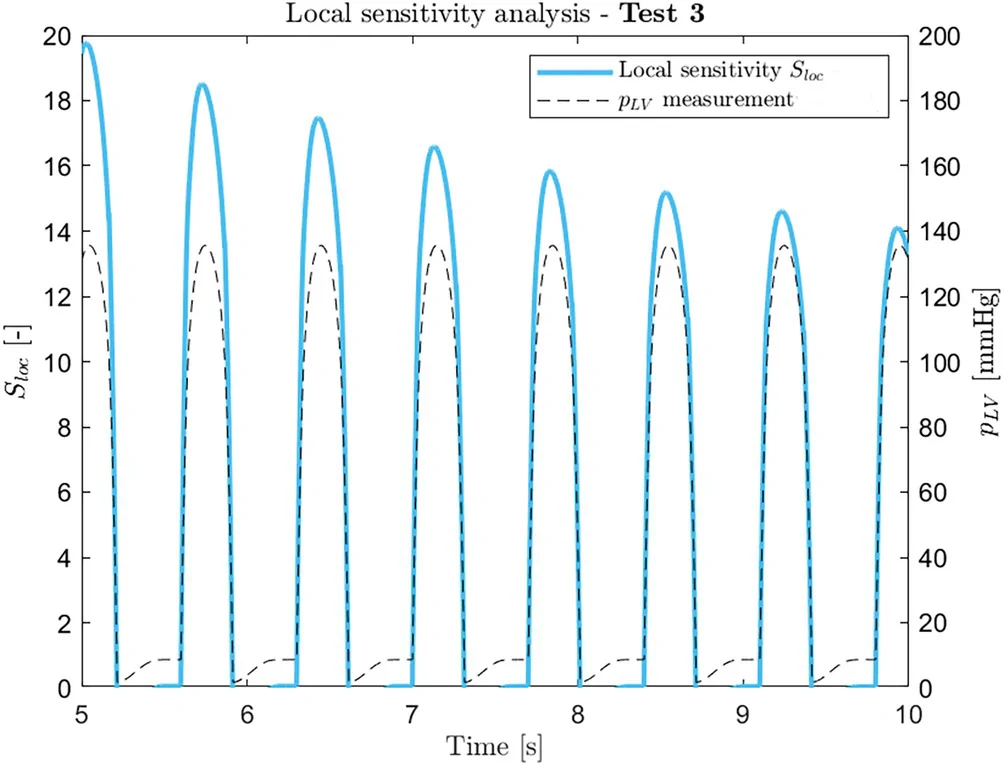
The local sensitivity $S_{\mathrm{loc}}$ plotted against the measured $p_{\mathrm{LV}}$ (Test 3).

First, a typical example of the local sensitivity analysis results is shown in Figure 5. In all test scenarios, the local sensitivity curve showed a similar pattern. The only variation between the tests was the amplitude of the local sensitivity $S_{\mathrm{loc}}$, which ranged from 5 to 35. The local sensitivity curve followed the same shape as the measured $p_{\mathrm{LV}}$ curve, showing non‐zero sensitivity only during the contraction phase of the cardiac cycle. Second, the results of the identifiability analysis are presented in Table 4, indicating that the CI is bounded in both directions for Tests 1 through 8, while being unbounded in one direction for Test 9. For Tests 1 through 6, the PL‐based CI (Table 4) is slightly wider than the PDF‐based CI (Table 2), whereas it is significantly wider for the tests with noisy measurement (Tests 7 through 9). Moreover, the PL $c_{\mathrm{LV}}$ (Table 4) differs from the estimated $c_{\mathrm{LV}}$ (Table 2) across all tests. This discrepancy remained minor in the tests without measurement noise (Test 1 to 6), but became larger as the level of measurement noise increased (Tests 7 to 9). Lastly, the stability of the mathematical model was evaluated across a range of $c_{\mathrm{LV}}$ values, specifically from 0 to 2.2 with a step size of 0.1. Based on this stability analysis, the equilibrium point ${\underset{˜}{x}}^{-}$ of the mathematical model was determined local asymptotically stable in a domain that reached from $c_{\mathrm{LV}}$ = 0.1 to $c_{\mathrm{LV}}$ = 2.0. The results of the stability analysis are given in Table C1 (Appendix C).

**TABLE 4:** Results of the identifiability analysis conducted on the synthetic data of Tests 1 through 9.

Test	True $c_{\mathrm{LV}}$ (‐)	Noise (%)	PL $c_{\mathrm{LV}}$ (‐)	PL CI $c_{\mathrm{LV}}$ (‐)	Diff. $c_{\mathrm{LV}}$ (‐)
1	0.4	—	0.406	[0.370, 0.442]	0.006
2	0.6	—	0.608	[0.567, 0.649]	0.008
3	0.8	—	0.813	[0.769, 0.857]	0.013
4	1.0	—	1.019	[0.973, 1.066]	0.019
5	1.2	—	1.227	[1.185, 1.275]	0.027
6	1.4	—	1.450	[1.417, 1.485]	0.050
7	0.8	10	0.733	[0.405, 1.321]	−0.067
8	0.8	20	0.670	[0.280, 2.130]	−0.130
9	0.8	50	0.483	[0.113, −]	0.317

*Note:* The $c_{\mathrm{LV}}$ calculated by executing the identifiability analysis is denoted as PL $c_{\mathrm{LV}}$, the belonging confidence interval as PL CI $c_{\mathrm{LV}}$, and the difference between the PL $c_{\mathrm{LV}}$ and true $c_{\mathrm{LV}}$ as Diff. $c_{\mathrm{LV}}$.

### 3.3 In Vivo Data

The dataset used consisted of 13 patients, since three patients were excluded due to technical limitations, and in one case it was not possible to execute the post‐TAVI measurement series for clinical reasons. Therefore, the dataset contained 13 pre‐TAVI and 12 post‐TAVI measurement series, a total of 25 measurement series to which the algorithm was successfully applied.

**FIGURE 6:**
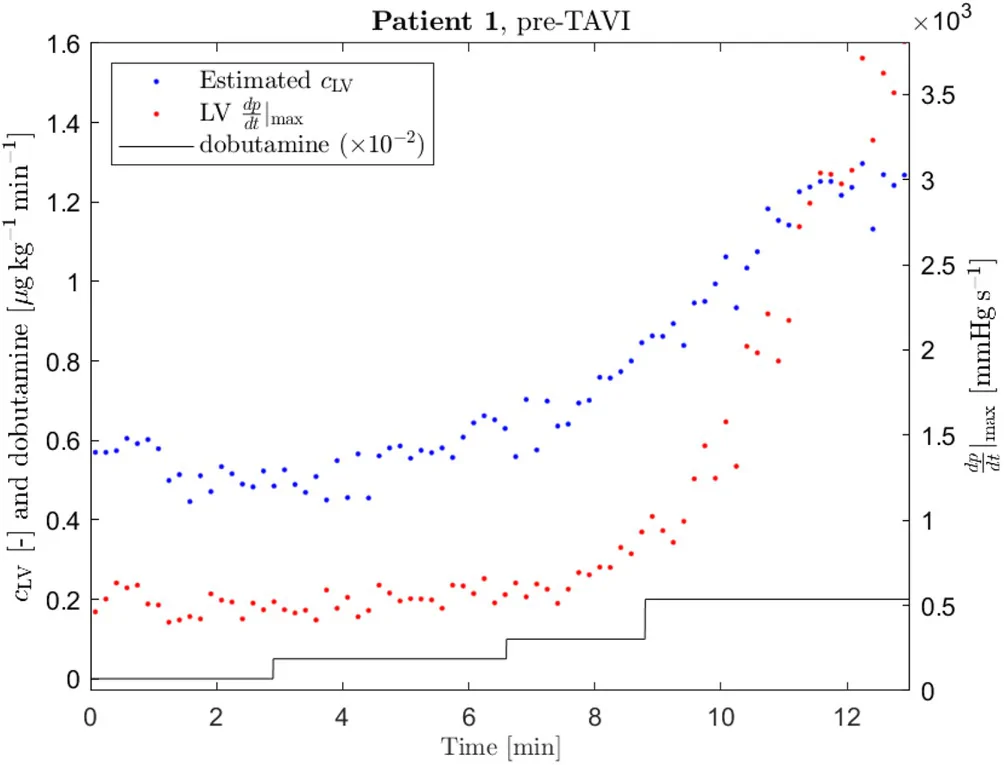
Illustration of the estimated $c_{\mathrm{LV}}$, the LV ${\left.\frac{\mathrm{dp}}{\mathrm{dt}}\right|}_{\max}$ and the administered dobutamine dose in patient 1, recorded before conducting the TAVI procedure.

**FIGURE 7:**
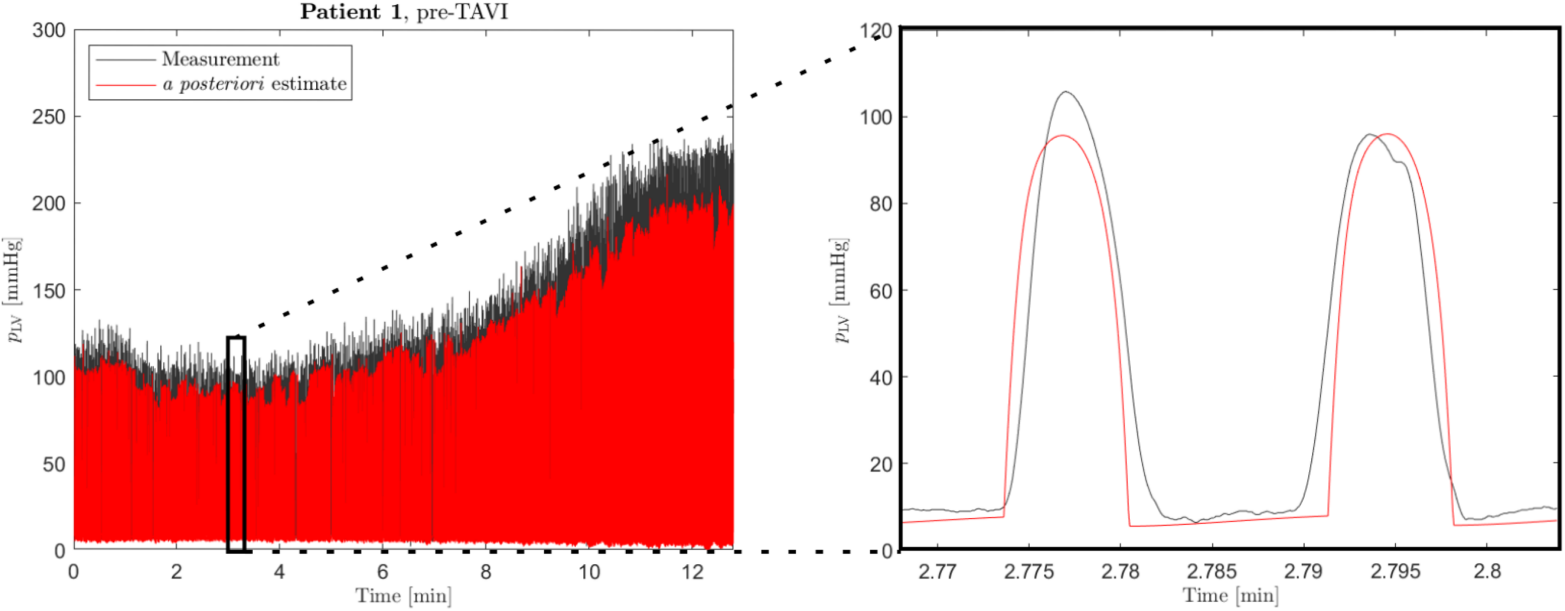
Comparison of the measured $p_{\mathrm{LV}}$ and the a posteriori estimated $p_{\mathrm{LV}}$ of patient 1 pre‐TAVI, with the full measurement interval depicted on the left and a shorter time scale displayed on the right.

Two different graphs were plotted, one showing the estimated $c_{\mathrm{LV}}$, the LV ${\left.\frac{\mathrm{dp}}{\mathrm{dt}}\right|}_{\max}$ and the stepwise dobutamine administration over time (Figure 6), and another comparing the measured $p_{\mathrm{LV}}$ and the a posteriori estimated $p_{\mathrm{LV}}$ over time (Figure 7).

First, based on the estimated $c_{\mathrm{LV}}$ and dobutamine data shown in Figure 6, it was evaluated whether the algorithm was able to identify the presumed increase in $c_{\mathrm{LV}}$ due to dobutamine administration. This increase in $c_{\mathrm{LV}}$ can be observed in Figure 6, however, it was not present in every measurement series of the in vivo dataset. Second, based on the left panel of Figure 7, the difference between the measured $p_{\mathrm{LV}}$ and the a posteriori estimated $p_{\mathrm{LV}}$ was assessed. The data in this figure illustrates that the shape of the a posteriori estimated $p_{\mathrm{LV}}$ is similar to that of the measured $p_{\mathrm{LV}}$, and that the a posteriori estimated $p_{\mathrm{LV}}$ is consistently lower than the measured $p_{\mathrm{LV}}$. These two observations were consistent across all measurement series in the dataset. Furthermore, the data in Figure 7 displays that the discrepancy between the measured $p_{\mathrm{LV}}$ and the a posteriori estimated $p_{\mathrm{LV}}$ depends on the amplitude of the measured $p_{\mathrm{LV}}$. Specifically, this discrepancy increases with higher measured $p_{\mathrm{LV}}$ values, with this observation being consistent throughout the dataset.

**FIGURE 8:**
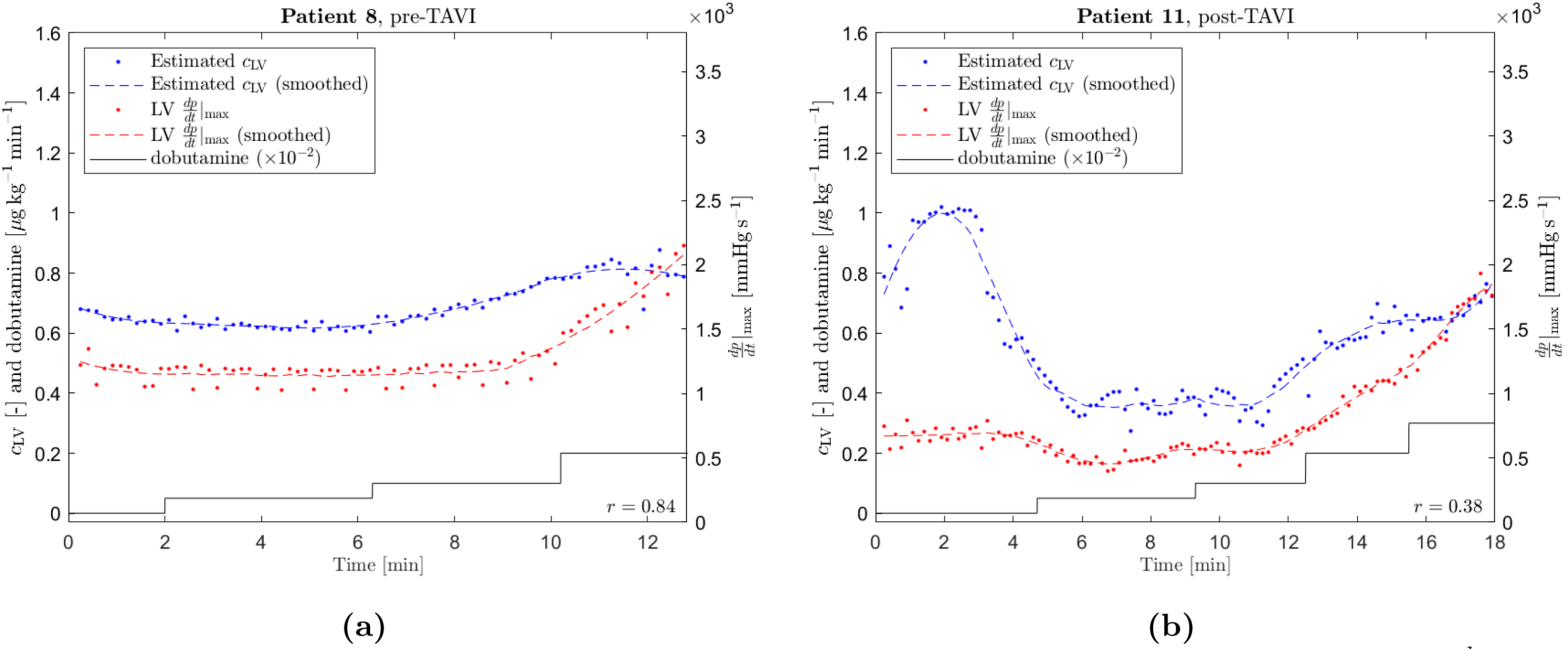
Two representative examples showing the estimated $c_{\mathrm{LV}}$, estimated $c_{\mathrm{LV}}$ (smoothed), LV ${\left.\frac{\mathrm{dp}}{\mathrm{dt}}\right|}_{\max}$, LV ${\left.\frac{\mathrm{dp}}{\mathrm{dt}}\right|}_{\max}$ (smoothed), and stepwise administered dobutamine over time. Panel (a) shows a strongly correlated measurement series (r ≥ 0.7), and panel (b) shows a moderate‐to‐weakly correlated measurement series (r < 0.7). Examples were selected as the measurement series with a r value closest to the median r within each category.

**FIGURE 9:**
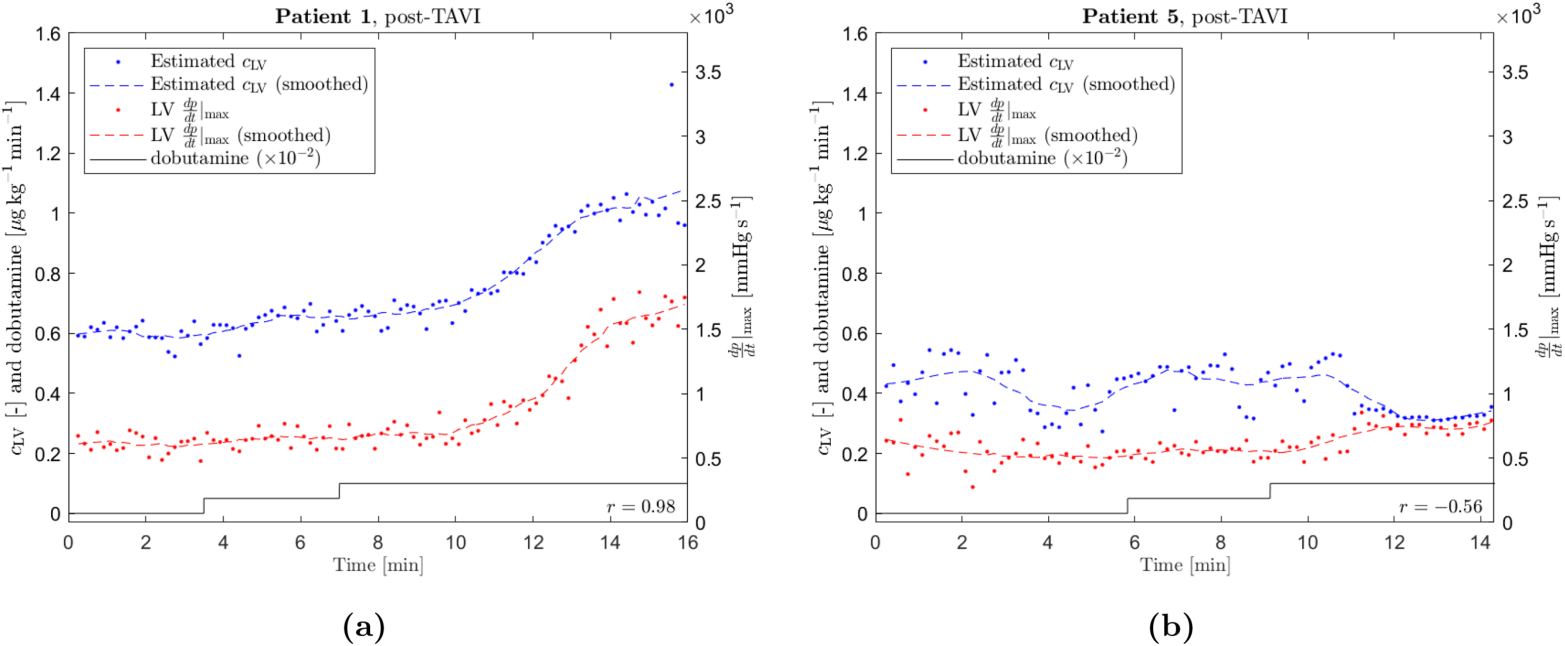
Two plots showing the estimated $c_{\mathrm{LV}}$, estimated $c_{\mathrm{LV}}$ (smoothed), LV ${\left.\frac{\mathrm{dp}}{\mathrm{dt}}\right|}_{\max}$, LV ${\left.\frac{\mathrm{dp}}{\mathrm{dt}}\right|}_{\max}$ (smoothed), and stepwise administered dobutamine over time. Panel (a) shows the measurement series with the highest observed r value, and panel (b) the measurement series with the lowest observed r value.

Third, for each measurement series in the dataset, the Pearson correlation coefficient (r) was calculated between the smoothed versions of the estimated $c_{\mathrm{LV}}$ and LV ${\left.\frac{\mathrm{dp}}{\mathrm{dt}}\right|}_{\max}$. Across all 25 measurement series, the correlation coefficient showed a median value of r=0.74 (IQR: 0.51–0.90; range: −0.66–0.98). Based on the predefined threshold, all measurement series in the dataset were classified as either strongly correlated (r ≥ 0.7) or moderate‐to‐weakly correlated (r < 0.7). In total, 16 out of 25 measurement series showed a strong correlation, whereas 9 out of 25 depicted a moderate‐to‐weak correlation. The full analysis of all measurement series, including the corresponding correlation coefficients, is provided in Supporting Information S1. Figure 8 shows a representative example for both categories, selected as the measurement series with a r value closest to the median r within each category. Additionally, Figure 9 presents the measurement series with the highest and lowest observed r value, illustrating the range of r values across the dataset. Strong and moderate‐to‐weak correlations were observed in both pre‐ and post‐TAVI measurement series. Despite the correlation classification, different temporal patterns and trends were observed within both categories.

## 4 Discussion

The aim of this study was to provide proof of principle for developing a ROUKF‐based DT (hereafter algorithm) for a human cardiovascular application, using a lumped‐parameter model of the systemic circulation of a heart failure patient. This algorithm was designed to estimate the LV contractility (hereafter parameter), which was evaluated using synthetic and in vivo LV pressure data as input. Three analyses were conducted using synthetic data: sensitivity, identifiability, and stability analyses. The proof of principle using synthetic data was considered successful when the algorithm provided real‐time parameter estimates with a narrow confidence interval, captured parameter changes, identified unique parameter values, and maintained mathematical model stability. The proof of principle under real‐world conditions was evaluated using in vivo data, and was deemed successful when the algorithm was capable of tracking cardiovascular state changes in real‐time.

Based on its application to synthetic data, the algorithm's ability to converge to the true parameter proved to be independent of the initialized parameter value, because convergence to the true parameter was observed in all test scenarios in which the initialized parameter value was kept identical. Also, its capability to converge was not affected by noisy measurements, since no significant deviations in the estimated parameter value were encountered when noise was added to the data. Then, the algorithm was capable of tracking parameter changes, even in scenarios with extreme (physiologically non‐feasible) parameter changes. Tracking parameter changes is a crucial capability of the algorithm since a patient's physiological state can change at any moment in time, for example due to physical exercise, administration of intravenous drugs or vascular intervention. The three mentioned characteristics: independence of initial condition, immunity to noisy measurements, and ability to track parameter changes confirm the algorithm's performance and contribute to the proof of principle.

The local sensitivity analysis depicted that the parameter was only sensitive for changes in the $p_{\mathrm{LV}}$ measurement during the contraction phase of the cardiac cycle. Second, the identifiability analysis showed a bounded confidence interval in both directions for almost all test scenarios. This implies that the parameter was identifiable when the data contained no or a limited level of noise, whereas the parameter became practically non‐identifiable when the noise level was too high. These findings are consistent with the previous research, because the measurement error gives rise to parameter uncertainty, which is subsequently causing practical non‐identifiability [35]. Third, the stability analysis revealed that the equilibrium point of the mathematical model was locally asymptotically stable in a parameter domain ranging from 0.1 to 2.0. This range was considered plausible for the envisioned cardiovascular application, since the parameter cannot be lower than approximately 0.3 to be viable, and can only be higher than approximately 1.5 in well‐trained athletes or in patients under inotropic support conditions, as stated in Section 2.5.2. Evidence of the algorithm's ability to identify unique parameter values, together with the stability of the mathematical model within a physiologically realistic range, contribute to demonstrating the algorithm's performance.

The application to in vivo data demonstrated that the algorithm was capable of using clinically acquired, patient‐specific measurements, as input. This is promising in terms of the algorithm's robustness, because these measurements are inherently more complex and may contain imperfections that are not present in synthetic data. These imperfections can be attributed to multiple factors, for example: the measurement equipment, the environment, the physiology of the patient or the clinician performing the measurement [47, 48]. Then, the algorithm was able to track increases in the parameter following dobutamine administration in several patients, supporting the proof of principle under real‐world conditions. Second, comparison of the a posteriori estimated $p_{\mathrm{LV}}$ and the measured $p_{\mathrm{LV}}$ showed a consistent shape alignment across all patients in the dataset. Third, in a considerable number of patients, the parameter exhibited a strong correlation with the surrogate marker for contractility, LV ${\left.\frac{\mathrm{dp}}{\mathrm{dt}}\right|}_{\max}$. Together, these two findings prove the algorithm's ability to capture changes in cardiovascular state. However, in some patients, the parameter and the surrogate marker showed a moderate‐to‐weak correlation, which may provide additional insight into patient‐specific cardiovascular conditions. Nevertheless, the algorithm's applicability to in vivo data provides evidence for the proof of principle under real‐world conditions, although further research is required to investigate the patients which showed a moderate‐to‐weak correlation between the parameter and the surrogate marker.

### 4.1 Limitations

In this study, the algorithm was applied to synthetic and in vivo data, whereas regulated in vitro examinations could be used to validate its performance, since the true parameter value is known in an in vitro setup. Nevertheless, providing proof of principle based on synthetic and in vivo data are both considered fundamental steps towards clinical application. Furthermore, the measurement error covariance matrix R, one of the initialization factors of the ROUKF algorithm (Appendix B), was arbitrarily chosen in this study. An accurate description of the noise statistics is crucial for the performance of the algorithm, as an incorrect description can lead to significantly degraded estimation accuracy or even algorithm failure [49]. Because matrix R was arbitrarily chosen in this study, estimation accuracy could degrade over time, whereas the degree of degradation is unknown. Ideally, it should have been adapted based on the trueness and precision of the used measurement equipment [50]. However, this was not feasible in this study, because the in vivo data were obtained from Johnson et al. [24]. Also, these noise statistics can be used for calculating the cost function of the identifiability analysis, leading to a more accurate determination of the confidence interval of the parameter, which is considered vital in clinical application [36]. Furthermore, the re‐initialization conditions were equal to the initialization conditions in this study, which were originally chosen to allocate the estimation error to the parameter space. This could distort the actual behavior of the estimation error over time, potentially causing valuable information about its development to be lost, whereas an accurate description of this error is considered indispensable in clinical practice.

The PL‐based identifiability analysis provided a significantly wider CI compared to the PDF‐based interval, indicating a possible overconfidence of the algorithm, as the PL‐based CI is considered more accurate. There are two primary reasons why the PL method is preferred over classical approaches, such as the PDF method, to determine the CI. First, in non‐linear models, confidence intervals can exhibit complex shapes due to the structure of the likelihood surface, and PL methods are inherently robust to such non‐linearities [51]. Second, the PL method does not require assumptions about the sampling distribution, unlike the PDF method, which is assumed to be log‐normally distributed in this study [36, 52]. These factors could explain the observed overconfidence. Another contributing factor could be the assumed rank reduction of the covariance matrix, which challenges the validity of the initialization and re‐initialization conditions. Furthermore, hybrid modeling approaches, such as the ROUKF, are established to enhance the accuracy of physics‐based models by using available data. However, these approaches are only suitable for short time‐scale approximations, while failing to capture long‐term parameter evolution, since underlying physiological mechanisms are often not precisely understood [53].

The in vivo dataset [24] does not contain values for the parameter of interest, resulting in an unknown difference between the estimated and the true parameter. Therefore, a surrogate marker for LV contractility, LV ${\left.\frac{\mathrm{dp}}{\mathrm{dt}}\right|}_{\max}$, was used to validate parameter estimates. However, the marker and the parameter remain intrinsically different, making accurate validation of algorithm's performance infeasible. Also, the model error is described as the discrepancy between the model and the physical reality [29], often quantified as model discrepancy, which was unknown in the current study. Ignoring model discrepancy can result in biased parameter predictions regardless of data quantity [54]. Additionally, model parameters were not tuned to patient characteristics, since the used dataset contained $p_{\mathrm{LV}}$ measurements only. Meiburg et al. displays that the UKF can be applied to estimate multiple parameters, which could be used to tune parameters of the mathematical model, but also to improve the algorithm's clinical utility. All abovementioned factors complicate the assessment of the patient‐specific results, implying that only generic conclusions can be drawn from the in vivo dataset.

The results of the in vivo application showed that in some patients, the estimated parameter did not increase when dobutamine was administered, which was contrary to previously established expectations. This could be attributable to the pathophysiological condition of these specific patients, because it is known that individuals with a stenotic aortic valve exhibit a reduced LV contractility [55]. Second, the comparison between the measured and the a posteriori estimated $p_{\mathrm{LV}}$ revealed that the algorithm systematically underestimated the measured $p_{\mathrm{LV}}$. Also, the discrepancy between these two quantities was dependent on the amplitude of the measured $p_{\mathrm{LV}}$. Both observations could result from not aligning the model parameters of the mathematical model with the patient characteristics of the dataset used, or from not accounting for model discrepancy. However, these observations could also be a result from using the computationally cheaper LO to estimate the states, which is known to be less accurate than the UKF. Either way, additional research is needed to investigate these claims. Ultimately, capturing the same behavior in the measured and the estimate $p_{\mathrm{LV}}$ is more important than obtaining identical values, as it reflects the adaptability to a patient‐specific scenario, which is vital for delivering proof of principle.

### 4.2 Future Work

Applying the algorithm to in vitro data would be a desirable direction for future research, as it allows the evaluation of the impact of model discrepancy, which is known to influence parameter uncertainty [54]. A possible approach for evaluating model discrepancy is to establish an in vitro setup that replicates the cardiovascular behavior described by the mathematical model used. This provides a configuration with zero model discrepancy, which can be used to validate the algorithm's performance. Subsequently, the in vitro setup can be gradually modified to deviate from the mathematical model, allowing the algorithm's performance to be evaluated under different levels of model discrepancy. Based on this discrepancy analysis, it can be evaluated whether the algorithm's overconfidence, as indicated by the identifiability analysis, is a genuine error or rather an invalid speculation. However, it remains challenging to establish an in vitro setup that accurately mimics the human cardiovascular circulation, while also allowing a stepwise evaluation of model discrepancy.

Regarding output processing, a method is desired that is able to describe every possible distribution of the PDF, leading to a possibly more truthful determination of the estimated parameter and its corresponding CI. Furthermore, a method capable of estimating the measurement error covariance matrix R could enhance the algorithm's performance, since accurate knowledge of noise statistics is essential for reliable parameter estimation [56]. This covariance matrix incorporates various stochastic components of a given system, like the instrumental and measurement function error [49]. However, these stochastic components can vary over time, making an estimator that can describe the evolution of the noise covariance matrix over time preferable. To identify the most suitable estimation method for this algorithm, future research needs be conducted.

The strength of the algorithm resides in its capability to predict cardiovascular states that would otherwise require invasive measurements or cannot be measured directly at all. Although this predictive capability was demonstrated on a single parameter in this study, it can be applied to a wide range of healthcare challenges. This is particularly valuable in cases where real‐time, patient‐specific information can support clinical decision‐making. To enable this, the mathematical model used in the ROUKF must be adapted to accurately describe the relevant physiological system for which the algorithm is intended. Ideally, this model should incorporate easily assessable data, as it would broaden the range of potential use cases and simplify the validation of the algorithm. Application to other healthcare challenges could prove the versatile potential of the algorithm, further highlighting its capability to personalize medicine and eventually improve patient outcomes.

## 5 Conclusion

We developed a DT for a human cardiovascular application using a ROUKF, capable of estimating LV contractility in real‐time, based on LV pressure data. When the algorithm was applied to synthetic data, it produced encouraging results and demonstrated strong performance. It converged to the true parameter value in all simulated scenarios, remained unaffected by noisy measurements, and its performance was independent of initial parameter conditions. Also, the estimated parameter proved to be identifiable, and the used mathematical model stable in a physiological feasible parameter range. Application to in vivo data demonstrated its robustness and ability to track cardiovascular state changes, supporting the proof of principle under real‐world conditions. However, questions were raised when the estimated parameter was compared to the conventional cardiac contractility parameter, requiring further research. To conclude, the ROUKF proved a promising method for the development of a DT for a human cardiovascular application. However, future work is required to further extract its potential and eventually prove the merit of using a DT for real‐time monitoring and personalizing medicine.

## Ethics Statement

The in vivo data used in the current study were obtained from Johnson et al. [24], https://doi.org/10.1093/eurheartj/ehy126. As described in Section 2.5.2, all subjects provided written informed consent and the study protocol was approved by the Medical Ethics Committee of the Catharina hospital (Eindhoven, The Netherlands). The current study involves the secondary use of previously collected data. These data were used in an anonymized form, and the authors of the current study had no contact with the participants. Furthermore, the dataset contained exclusively raw measurement signals and did not include any supplementary metadata. Therefore, no additional ethical approval was required.

## Conflicts of Interest

The authors declare no conflicts of interest.

## Supporting information


**Figure S1:** Estimated $c_{\mathrm{LV}}$, estimated $c_{\mathrm{LV}}$ (smoothed), LV ${\left.\frac{\mathrm{dp}}{\mathrm{dt}}\right|}_{\max}$, LV ${\left.\frac{\mathrm{dp}}{\mathrm{dt}}\right|}_{\max}$ (smoothed), and stepwise administered dobutamine depicted over time in Patient 1 before TAVI (pre‐TAVI). The Pearson correlation coefficient between estimated $c_{\mathrm{LV}}$ (smoothed) and LV ${\left.\frac{\mathrm{dp}}{\mathrm{dt}}\right|}_{\max}$ (smoothed) was r=0.94, classified as strongly correlated (r ≥ 0.7).
**Figure S2:** Estimated $c_{\mathrm{LV}}$, estimated $c_{\mathrm{LV}}$ (smoothed), LV ${\left.\frac{\mathrm{dp}}{\mathrm{dt}}\right|}_{\max}$, LV ${\left.\frac{\mathrm{dp}}{\mathrm{dt}}\right|}_{\max}$ (smoothed), and stepwise administered dobutamine depicted over time in Patient 1 after TAVI (post‐TAVI). The Pearson correlation coefficient between estimated $c_{\mathrm{LV}}$ (smoothed) and LV ${\left.\frac{\mathrm{dp}}{\mathrm{dt}}\right|}_{\max}$ (smoothed) was r=0.98, classified as strongly correlated (r ≥ 0.7).
**Figure S3:** Estimated $c_{\mathrm{LV}}$, estimated $c_{\mathrm{LV}}$ (smoothed), LV ${\left.\frac{\mathrm{dp}}{\mathrm{dt}}\right|}_{\max}$, LV ${\left.\frac{\mathrm{dp}}{\mathrm{dt}}\right|}_{\max}$ (smoothed), and stepwise administered dobutamine depicted over time in Patient 2 before TAVI (pre‐TAVI). The Pearson correlation coefficient between estimated $c_{\mathrm{LV}}$ (smoothed) and LV ${\left.\frac{\mathrm{dp}}{\mathrm{dt}}\right|}_{\max}$ (smoothed) was r=0.81, classified as strongly correlated (r ≥ 0.7).
**Figure S4:** Estimated $c_{\mathrm{LV}}$, estimated $c_{\mathrm{LV}}$ (smoothed), LV ${\left.\frac{\mathrm{dp}}{\mathrm{dt}}\right|}_{\max}$, LV ${\left.\frac{\mathrm{dp}}{\mathrm{dt}}\right|}_{\max}$ (smoothed), and stepwise administered dobutamine depicted over time in Patient 2 after TAVI (post‐TAVI). The Pearson correlation coefficient between estimated $c_{\mathrm{LV}}$ (smoothed) and LV ${\left.\frac{\mathrm{dp}}{\mathrm{dt}}\right|}_{\max}$ (smoothed) was r=0.90, classified as strongly correlated (r ≥ 0.7).
**Figure S5:** Estimated $c_{\mathrm{LV}}$, estimated $c_{\mathrm{LV}}$ (smoothed), LV ${\left.\frac{\mathrm{dp}}{\mathrm{dt}}\right|}_{\max}$, LV ${\left.\frac{\mathrm{dp}}{\mathrm{dt}}\right|}_{\max}$ (smoothed), and stepwise administered dobutamine depicted over time in Patient 3 before TAVI (pre‐TAVI). The Pearson correlation coefficient between estimated $c_{\mathrm{LV}}$ (smoothed) and LV ${\left.\frac{\mathrm{dp}}{\mathrm{dt}}\right|}_{\max}$ (smoothed) was r=−0.66, classified as moderate‐to‐weakly correlated (r < 0.7).
**Figure S6:** Estimated $c_{\mathrm{LV}}$, estimated $c_{\mathrm{LV}}$ (smoothed), LV ${\left.\frac{\mathrm{dp}}{\mathrm{dt}}\right|}_{\max}$, LV ${\left.\frac{\mathrm{dp}}{\mathrm{dt}}\right|}_{\max}$ (smoothed), and stepwise administered dobutamine depicted over time in Patient 3 after TAVI (post‐TAVI). The Pearson correlation coefficient between estimated $c_{\mathrm{LV}}$ (smoothed) and LV ${\left.\frac{\mathrm{dp}}{\mathrm{dt}}\right|}_{\max}$ (smoothed) was r=0.74, classified as strongly correlated (r ≥ 0.7).
**Figure S7:** Estimated $c_{\mathrm{LV}}$, estimated $c_{\mathrm{LV}}$ (smoothed), LV ${\left.\frac{\mathrm{dp}}{\mathrm{dt}}\right|}_{\max}$, LV ${\left.\frac{\mathrm{dp}}{\mathrm{dt}}\right|}_{\max}$ (smoothed), and stepwise administered dobutamine depicted over time in Patient 4 before TAVI (pre‐TAVI). The Pearson correlation coefficient between estimated $c_{\mathrm{LV}}$ (smoothed) and LV ${\left.\frac{\mathrm{dp}}{\mathrm{dt}}\right|}_{\max}$ (smoothed) was r=0.96, classified as strongly correlated (r ≥ 0.7).
**Figure S8:** Estimated $c_{\mathrm{LV}}$, estimated $c_{\mathrm{LV}}$ (smoothed), LV ${\left.\frac{\mathrm{dp}}{\mathrm{dt}}\right|}_{\max}$, LV ${\left.\frac{\mathrm{dp}}{\mathrm{dt}}\right|}_{\max}$ (smoothed), and stepwise administered dobutamine depicted over time in Patient 4 after TAVI (post‐TAVI). The Pearson correlation coefficient between estimated $c_{\mathrm{LV}}$ (smoothed) and LV ${\left.\frac{\mathrm{dp}}{\mathrm{dt}}\right|}_{\max}$ (smoothed) was r=0.27, classified as moderate‐to‐weakly correlated (r < 0.7).
**Figure S9:** Estimated $c_{\mathrm{LV}}$, estimated $c_{\mathrm{LV}}$ (smoothed), LV ${\left.\frac{\mathrm{dp}}{\mathrm{dt}}\right|}_{\max}$, LV ${\left.\frac{\mathrm{dp}}{\mathrm{dt}}\right|}_{\max}$ (smoothed), and stepwise administered dobutamine depicted over time in Patient 5 before TAVI (pre‐TAVI). The Pearson correlation coefficient between estimated $c_{\mathrm{LV}}$ (smoothed) and LV ${\left.\frac{\mathrm{dp}}{\mathrm{dt}}\right|}_{\max}$ (smoothed) was r=0.70, classified as strongly correlated (r ≥ 0.7).
**Figure S10:** Estimated $c_{\mathrm{LV}}$, estimated $c_{\mathrm{LV}}$ (smoothed), LV ${\left.\frac{\mathrm{dp}}{\mathrm{dt}}\right|}_{\max}$, LV ${\left.\frac{\mathrm{dp}}{\mathrm{dt}}\right|}_{\max}$ (smoothed), and stepwise administered dobutamine depicted over time in Patient 5 after TAVI (post‐TAVI). The Pearson correlation coefficient between estimated $c_{\mathrm{LV}}$ (smoothed) and LV ${\left.\frac{\mathrm{dp}}{\mathrm{dt}}\right|}_{\max}$ (smoothed) was r=−0.56, classified as moderate‐to‐weakly correlated (r < 0.7).
**Figure S11:** Estimated $c_{\mathrm{LV}}$, estimated $c_{\mathrm{LV}}$ (smoothed), LV ${\left.\frac{\mathrm{dp}}{\mathrm{dt}}\right|}_{\max}$, LV ${\left.\frac{\mathrm{dp}}{\mathrm{dt}}\right|}_{\max}$ (smoothed), and stepwise administered dobutamine depicted over time in Patient 6 before TAVI (pre‐TAVI). The Pearson correlation coefficient between estimated $c_{\mathrm{LV}}$ (smoothed) and LV ${\left.\frac{\mathrm{dp}}{\mathrm{dt}}\right|}_{\max}$ (smoothed) was r=0.55, classified as moderate‐to‐weakly correlated (r < 0.7).
**Figure S12:** Estimated $c_{\mathrm{LV}}$, estimated $c_{\mathrm{LV}}$ (smoothed), LV ${\left.\frac{\mathrm{dp}}{\mathrm{dt}}\right|}_{\max}$, LV ${\left.\frac{\mathrm{dp}}{\mathrm{dt}}\right|}_{\max}$ (smoothed), and stepwise administered dobutamine depicted over time in Patient 6 after TAVI (post‐TAVI). The Pearson correlation coefficient between estimated $c_{\mathrm{LV}}$ (smoothed) and LV ${\left.\frac{\mathrm{dp}}{\mathrm{dt}}\right|}_{\max}$ (smoothed) was r=0.51, classified as moderate‐to‐weakly correlated (r < 0.7).
**Figure S13:** Estimated $c_{\mathrm{LV}}$, estimated $c_{\mathrm{LV}}$ (smoothed), LV ${\left.\frac{\mathrm{dp}}{\mathrm{dt}}\right|}_{\max}$, LV ${\left.\frac{\mathrm{dp}}{\mathrm{dt}}\right|}_{\max}$ (smoothed), and stepwise administered dobutamine depicted over time in Patient 7 before TAVI (pre‐TAVI). The Pearson correlation coefficient between estimated $c_{\mathrm{LV}}$ (smoothed) and LV ${\left.\frac{\mathrm{dp}}{\mathrm{dt}}\right|}_{\max}$ (smoothed) was r=0.79, classified as strongly correlated (r ≥ 0.7).
**Figure S14:** Estimated $c_{\mathrm{LV}}$, estimated $c_{\mathrm{LV}}$ (smoothed), LV ${\left.\frac{\mathrm{dp}}{\mathrm{dt}}\right|}_{\max}$, LV ${\left.\frac{\mathrm{dp}}{\mathrm{dt}}\right|}_{\max}$ (smoothed), and stepwise administered dobutamine depicted over time in Patient 7 after TAVI (post‐TAVI). The Pearson correlation coefficient between estimated $c_{\mathrm{LV}}$ (smoothed) and LV ${\left.\frac{\mathrm{dp}}{\mathrm{dt}}\right|}_{\max}$ (smoothed) was r=0.92, classified as strongly correlated (r ≥ 0.7).
**Figure S15:** Estimated $c_{\mathrm{LV}}$, estimated $c_{\mathrm{LV}}$ (smoothed), LV ${\left.\frac{\mathrm{dp}}{\mathrm{dt}}\right|}_{\max}$, LV ${\left.\frac{\mathrm{dp}}{\mathrm{dt}}\right|}_{\max}$ (smoothed), and stepwise administered dobutamine depicted over time in Patient 8 before TAVI (pre‐TAVI). The Pearson correlation coefficient between estimated $c_{\mathrm{LV}}$ (smoothed) and LV ${\left.\frac{\mathrm{dp}}{\mathrm{dt}}\right|}_{\max}$ (smoothed) was r=0.84, classified as strongly correlated (r ≥ 0.7).
**Figure S16:** Estimated $c_{\mathrm{LV}}$, estimated $c_{\mathrm{LV}}$ (smoothed), LV ${\left.\frac{\mathrm{dp}}{\mathrm{dt}}\right|}_{\max}$, LV ${\left.\frac{\mathrm{dp}}{\mathrm{dt}}\right|}_{\max}$ (smoothed), and stepwise administered dobutamine depicted over time in Patient 8 after TAVI (post‐TAVI). The Pearson correlation coefficient between estimated $c_{\mathrm{LV}}$ (smoothed) and LV ${\left.\frac{\mathrm{dp}}{\mathrm{dt}}\right|}_{\max}$ (smoothed) was r=0.50, classified as moderate‐to‐weakly correlated (r < 0.7).
**Figure S17:** Estimated $c_{\mathrm{LV}}$, estimated $c_{\mathrm{LV}}$ (smoothed), LV ${\left.\frac{\mathrm{dp}}{\mathrm{dt}}\right|}_{\max}$, LV ${\left.\frac{\mathrm{dp}}{\mathrm{dt}}\right|}_{\max}$ (smoothed), and stepwise administered dobutamine depicted over time in Patient 9 before TAVI (pre‐TAVI). The Pearson correlation coefficient between estimated $c_{\mathrm{LV}}$ (smoothed) and LV ${\left.\frac{\mathrm{dp}}{\mathrm{dt}}\right|}_{\max}$ (smoothed) was r=0.73, classified as strongly correlated (r ≥ 0.7).
**Figure S18:** Estimated $c_{\mathrm{LV}}$, estimated $c_{\mathrm{LV}}$ (smoothed), LV ${\left.\frac{\mathrm{dp}}{\mathrm{dt}}\right|}_{\max}$, LV ${\left.\frac{\mathrm{dp}}{\mathrm{dt}}\right|}_{\max}$ (smoothed), and stepwise administered dobutamine depicted over time in Patient 9 after TAVI (post‐TAVI). The Pearson correlation coefficient between estimated $c_{\mathrm{LV}}$ (smoothed) and LV ${\left.\frac{\mathrm{dp}}{\mathrm{dt}}\right|}_{\max}$ (smoothed) was r=0.83, classified as strongly correlated (r ≥ 0.7).
**Figure S19:** Estimated $c_{\mathrm{LV}}$, estimated $c_{\mathrm{LV}}$ (smoothed), LV ${\left.\frac{\mathrm{dp}}{\mathrm{dt}}\right|}_{\max}$, LV ${\left.\frac{\mathrm{dp}}{\mathrm{dt}}\right|}_{\max}$ (smoothed), and stepwise administered dobutamine depicted over time in Patient 10 before TAVI (pre‐TAVI). The Pearson correlation coefficient between estimated $c_{\mathrm{LV}}$ (smoothed) and LV ${\left.\frac{\mathrm{dp}}{\mathrm{dt}}\right|}_{\max}$ (smoothed) was r=0.88, classified as strongly correlated (r ≥ 0.7).
**Figure S20:** Estimated $c_{\mathrm{LV}}$, estimated $c_{\mathrm{LV}}$ (smoothed), LV ${\left.\frac{\mathrm{dp}}{\mathrm{dt}}\right|}_{\max}$, LV ${\left.\frac{\mathrm{dp}}{\mathrm{dt}}\right|}_{\max}$ (smoothed), and stepwise administered dobutamine depicted over time in Patient 11 before TAVI (pre‐TAVI). The Pearson correlation coefficient between estimated $c_{\mathrm{LV}}$ (smoothed) and LV ${\left.\frac{\mathrm{dp}}{\mathrm{dt}}\right|}_{\max}$ (smoothed) was r=0.90, classified as strongly correlated (r ≥ 0.7).
**Figure S21:** Estimated $c_{\mathrm{LV}}$, estimated $c_{\mathrm{LV}}$ (smoothed), LV ${\left.\frac{\mathrm{dp}}{\mathrm{dt}}\right|}_{\max}$, LV ${\left.\frac{\mathrm{dp}}{\mathrm{dt}}\right|}_{\max}$ (smoothed), and stepwise administered dobutamine depicted over time in Patient 11 after TAVI (post‐TAVI). The Pearson correlation coefficient between estimated $c_{\mathrm{LV}}$ (smoothed) and LV ${\left.\frac{\mathrm{dp}}{\mathrm{dt}}\right|}_{\max}$ (smoothed) was r=0.38, classified as moderate‐to‐weakly correlated (r < 0.7).
**Figure S22:** Estimated $c_{\mathrm{LV}}$, estimated $c_{\mathrm{LV}}$ (smoothed), LV ${\left.\frac{\mathrm{dp}}{\mathrm{dt}}\right|}_{\max}$, LV ${\left.\frac{\mathrm{dp}}{\mathrm{dt}}\right|}_{\max}$ (smoothed), and stepwise administered dobutamine depicted over time in Patient 13 before TAVI (pre‐TAVI). The Pearson correlation coefficient between estimated $c_{\mathrm{LV}}$ (smoothed) and LV ${\left.\frac{\mathrm{dp}}{\mathrm{dt}}\right|}_{\max}$ (smoothed) was r=0.72, classified as strongly correlated (r ≥ 0.7).
**Figure S23:** Estimated $c_{\mathrm{LV}}$, estimated $c_{\mathrm{LV}}$ (smoothed), LV ${\left.\frac{\mathrm{dp}}{\mathrm{dt}}\right|}_{\max}$, LV ${\left.\frac{\mathrm{dp}}{\mathrm{dt}}\right|}_{\max}$ (smoothed), and stepwise administered dobutamine depicted over time in Patient 13 after TAVI (post‐TAVI). The Pearson correlation coefficient between estimated $c_{\mathrm{LV}}$ (smoothed) and LV ${\left.\frac{\mathrm{dp}}{\mathrm{dt}}\right|}_{\max}$ (smoothed) was r=−0.22, classified as moderate‐to‐weakly correlated (r < 0.7).
**Figure S24:** Estimated $c_{\mathrm{LV}}$, estimated $c_{\mathrm{LV}}$ (smoothed), LV ${\left.\frac{\mathrm{dp}}{\mathrm{dt}}\right|}_{\max}$, LV ${\left.\frac{\mathrm{dp}}{\mathrm{dt}}\right|}_{\max}$ (smoothed), and stepwise administered dobutamine depicted over time in Patient 14 before TAVI (pre‐TAVI). The Pearson correlation coefficient between estimated $c_{\mathrm{LV}}$ (smoothed) and LV ${\left.\frac{\mathrm{dp}}{\mathrm{dt}}\right|}_{\max}$ (smoothed) was r=0.93, classified as strongly correlated (r ≥ 0.7).
**Figure S25:** Estimated $c_{\mathrm{LV}}$, estimated $c_{\mathrm{LV}}$ (smoothed), LV ${\left.\frac{\mathrm{dp}}{\mathrm{dt}}\right|}_{\max}$, LV ${\left.\frac{\mathrm{dp}}{\mathrm{dt}}\right|}_{\max}$ (smoothed), and stepwise administered dobutamine depicted over time in Patient 14 after TAVI (post‐TAVI). The Pearson correlation coefficient between estimated $c_{\mathrm{LV}}$ (smoothed) and LV ${\left.\frac{\mathrm{dp}}{\mathrm{dt}}\right|}_{\max}$ (smoothed) was r=0.55, classified as moderate‐to‐weakly correlated (r < 0.7).

## Data Availability

Research data are not shared.

## Appendix A

The model used to describe the behavior of the human cardiovascular system originates from Meiburg et al. [29]. The set of governing equations $\underset{˜}{f}\left({\underset{˜}{x}}_{\mathrm{aug},k}\right)$, collectively representing the mathematical model, are casted to $\underset{˜}{g}\left({\underset{˜}{x}}_{\mathrm{aug},k}\right)$ = $\underset{˜}{0}$, and solved using the Newton–Raphson method. In this method, the inverse Jacobian of $\underset{˜}{g}\left({\underset{˜}{x}}_{\mathrm{aug},k}\right)$, denoted as $J_{\underset{˜}{g}}^{-1}\left({\underset{˜}{x}}_{\mathrm{aug},k}\right)$, is used to iteratively solve $\underset{˜}{g}\left({\underset{˜}{x}}_{\mathrm{aug},k}\right)$ = $\underset{˜}{0}$, which is exploited to update the augmented state vector ${\underset{˜}{x}}_{\mathrm{aug},k}$ of the current time step k to approximate ${\underset{˜}{x}}_{\mathrm{aug},k+1}$ of the next time step k+1:

(A1)
$${\underset{˜}{x}}_{\mathrm{aug},k+1}={\underset{˜}{x}}_{\mathrm{aug},k}-J_{\underset{˜}{g}}^{-1}\left({\underset{˜}{x}}_{\mathrm{aug},k}\right)\underset{˜}{g}\left({\underset{˜}{x}}_{\mathrm{aug},k}\right),$$

with $\underset{˜}{g}\left({\underset{˜}{x}}_{\mathrm{aug},k}\right)=0$ being represented as follows:

(A2)
$$g_{p_{\mathrm{LV}}}=p_{\mathrm{LV}}\left(t\right)-p_{\mathrm{LV}}\left(t+\Delta t\right)=0,$$

(A3)
$$g_{p_{\mathrm{ao}}}=p_{\mathrm{ao}}\left(t\right)-\frac{V_{c_{1}}}{C_{\mathrm{art}}}-q_{\mathrm{av}}\left(t\right)R_{\text{prox}}-\frac{L_{\mathrm{art}}}{\mathrm{dt}}q_{\mathrm{av}}\left(t\right)=0,$$

(A4)
$$g_{V_{\mathrm{LV}}}=V_{\mathrm{LV}}\left(t\right)-V_{\mathrm{LV}}\left(t-\Delta t\right)-q_{\mathrm{mv}}\left(t\right)\mathrm{dt}+q_{\mathrm{av}}\left(t\right)\mathrm{dt}=0,$$

(A5)
$$g_{q_{\mathrm{mv}}}=q_{\mathrm{mv}}\left(t\right)\frac{B_{\mathrm{mv}}+L_{\mathrm{mv}}}{\mathrm{dt}}-q_{\mathrm{mv}}\left(t-\Delta t\right)\frac{L_{\mathrm{mv}}}{\mathrm{dt}}-p_{\mathrm{pul}}+p_{\mathrm{LV}}\left(t\right)=0,$$

(A6)
$$\begin{array}{ll} g_{q_{\mathrm{av}}} & =B_{\mathrm{av}}q_{\mathrm{av}}\left(t\right)|q_{\mathrm{av}}\left(t\right)|+R_{\text{prox}}q_{\mathrm{av}}\left(t\right)+\frac{L_{\mathrm{art}}}{\mathrm{dt}}q_{\mathrm{av}}\left(t\right)\\ & \;-\frac{L_{\mathrm{art}}}{\mathrm{dt}}q_{\mathrm{av}}\left(t-\Delta t\right)-p_{\mathrm{LV}}+\frac{V_{c_{1}}}{C_{\mathrm{art}}}=0, \end{array}$$

(A7)
$$g_{V_{c_{1}}}=V_{c_{1}}\left(t\right)-V_{c_{1}}\left(t-\Delta t\right)-q_{\mathrm{av}}\left(t\right)\mathrm{dt}+q_{c_{1}}\left(t\right)\mathrm{dt}=0,$$

(A8)
$$g_{q_{c_{1}-c_{2}}}=q_{c_{1}-c_{2}}\left(t\right)\frac{R_{\text{prox}}+L_{\mathrm{art}}}{\mathrm{dt}}-q_{c_{1}-c_{2}}\left(t-\Delta t\right)\frac{L_{\mathrm{art}}}{\mathrm{dt}}-\frac{V_{c_{1}}\left(t\right)-V_{c_{2}}\left(t\right)}{C_{\mathrm{art}}}=0,$$

(A9)
$$g_{V_{c_{2}}}=V_{c_{1}}\left(t\right)-V_{c_{1}}\left(t-\Delta t\right)-q_{c_{1}-c_{2}}\left(t\right)\mathrm{dt}+q_{\mathrm{per}}\left(t\right)\mathrm{dt}=0,$$

(A10)
$$g_{q_{\mathrm{per}}}=q_{\mathrm{per}}\left(t\right)-\frac{V_{c_{2}}\left(t\right)}{C_{\mathrm{art}}R_{\text{dist}}}-\frac{p_{\mathrm{per}}}{R_{\text{dist}}}=0.$$

For initialization of the ROUKF algorithm, the initial a posteriori estimate ${\underset{˜}{\hat{x}}}_{\mathrm{aug},k=0}^{a}$ was arbitrarily defined. This augmented state vector ${\underset{˜}{x}}_{\mathrm{aug}}$ $\in {\mathbb{R}}^{n}$ consists of states $\underset{˜}{x}$ $\in {\mathbb{R}}^{n_{s}}$ and parameters $\underset{˜}{\theta }$ $\in {\mathbb{R}}^{n_{p}}$, with $n_{s}=9$, $n_{p}=1$, and $n=n_{s}+n_{p}=10$. In the current study, the parameter of interest θ is the left ventricular contractility $c_{\mathrm{LV}}$. The initial a posteriori estimate ${\underset{˜}{\hat{x}}}_{\mathrm{aug},k=0}^{a}$ can be expressed as follows:

(A11)
$${\underset{˜}{\hat{x}}}_{\mathrm{aug},0}^{a}=\left[\begin{array}{c} {\underset{˜}{\hat{x}}}_{0}\\ {\hat{\theta }}_{0} \end{array}\right]=\left[\begin{array}{c} p_{\mathrm{LV}}\\ p_{\mathrm{ao}}\\ V_{\mathrm{LV}}\\ q_{\mathrm{mv}}\\ q_{\mathrm{av}}\\ V_{c_{1}}\\ q_{c_{1}-c_{2}}\\ V_{c_{2}}\\ q_{\mathrm{per}}\\ c_{\mathrm{lv}} \end{array}\right]=\left[\begin{array}{cc} 8 & \left[\text{mmHg}\right]\\ 80 & \left[\text{mmHg}\right]\\ 2V_{\mathrm{LV},0} & \left[\mathrm{mL}\right]\\ 0 & \left[\frac{\mathrm{mL}}{s}\right]\\ 0 & \left[\frac{\mathrm{mL}}{s}\right]\\ p_{\mathrm{ao}}C_{\mathrm{art}} & \left[\mathrm{mL}\right]\\ 0 & \left[\frac{\mathrm{mL}}{s}\right]\\ p_{\mathrm{ao}}C_{\mathrm{art}} & \left[\mathrm{mL}\right]\\ 0 & \left[\frac{\mathrm{mL}}{s}\right]\\ 0.8 & \left[-\right] \end{array}\right].$$

According to Meiburg et al. [29], the unloaded cavity volume $V_{\mathrm{LV},0}$ was set to 80 mL and the arterial compliance $C_{\mathrm{art}}$ to 1 $\frac{\mathrm{mL}}{\text{mmHg}}$. Furthermore, the aortic valve resistance $B_{\mathrm{av}}$ was altered in the current study and set to $10^{-4}$ mmHg $s^{2}$ ${\mathrm{mL}}^{-2}$, because the aortic valve was assumed to be non‐stenotic.

## Appendix B

The ROUKF algorithm consists of three recursive steps: (1) sigma‐point determination, (2) prediction and (3) analysis, complemented with an initialization step that is conducted before the first iteration. The four steps are elaborated below, with the algorithm being indexed using discrete time step k.

### Initialization

The following non‐variant elements were defined in the initialization step: simplex sigma‐point matrix S, weight ε, diagonal matrix $D_{\varepsilon }$, measurement error covariance R, and non‐variant part of the covariance matrix $P_{\varepsilon }$. The definitions of S and ε are given in the Subsection 2.2.2, with R and $P_{\varepsilon }$ being arbitrarily set to: R=100·I and $P_{\varepsilon }$ = ${\mathrm{SD}}_{\varepsilon }S^{T}$. Furthermore, the initial a posteriori estimate ${\underset{˜}{\hat{x}}}_{\mathrm{aug},k=0}^{a}$ is given in Appendix A, with the initial conditions for the Luenberger matrix $L_{k=0}$ and the uncertainty matrix $U_{k=0}$ being expressed in Equation (9).

### Sigma‐Point Determination

In this step, the Cholesky matrix $C_{k}$ is determined using the Cholesky decomposition. Also, the sigma‐points of the current time step k are determined, with ${\underset{˜}{\chi }}_{i,k}$ denoting the *i*th column vector of the sigma‐point matrix $\chi_{k}$:

(A12)
$$C_{k}=\sqrt{U_{k}^{-1}},$$

(A13)
$${\underset{˜}{\chi }}_{i,k}={\underset{˜}{\hat{x}}}_{\mathrm{aug},k}^{a}+L_{k}C_{k}^{T}{\underset{˜}{S}}_{i},\;1\leq i\leq n_{p}+1.$$

### Prediction

First, the sigma‐points of the current time step $\chi_{k}$ are propagated to the prediction space of the next time step k+1 using the non‐linear transition function $\underset{˜}{f}\left({\underset{˜}{\chi }}_{i,k}\right)$. Next, the a priori estimate ${\underset{˜}{\hat{x}}}_{\mathrm{aug},k+1}^{p}$ and the Luenberger matrix $L_{k+1}$, both of the next time step k+1, are determined:

(A14)
$${\underset{˜}{\chi }}_{i,k+1}^{p}=\underset{˜}{f}\left({\underset{˜}{\chi }}_{i,k}\right),\;1\leq i\leq n_{p}+1,$$

(A15)
$${\underset{˜}{\hat{x}}}_{\mathrm{aug},k+1}^{p}=\sum_{i=1}^{n_{p}+1}\varepsilon {\underset{˜}{\chi }}_{i,k+1}^{p},$$

(A16)
$$L_{k+1}=\chi_{k+1}^{p}D_{\varepsilon }S^{T}.$$

### Analysis

During the final step, the a priori estimate ${\underset{˜}{\hat{x}}}_{\mathrm{aug},k+1}^{p}$ is updated using the measurements ${\underset{˜}{y}}_{k+1}$, resulting in the a posteriori estimate ${\underset{˜}{\hat{x}}}_{\mathrm{aug},k+1}^{a}$. First of all, the sigma‐points located in the prediction space $\chi_{k+1}^{p}$ will be propagated to the measurement space using the measurement function $\underset{˜}{h}$($\chi_{k+1}^{p}$). Subsequently, measurement space estimate ${\underset{˜}{\hat{z}}}_{k+1}$, the Luenberger matrix in the measurement space $B_{k+1}$ and the uncertainty matrix $U_{k+1}$ are determined before calculating the a posteriori covariance matrix $P_{k+1}^{a}$. Lastly, the a priori estimate ${\underset{˜}{\hat{x}}}_{\mathrm{aug},k+1}^{p}$ is updated using the weighted discrepancy between the measurement ${\underset{˜}{y}}_{k+1}$ and the measurement estimate ${\underset{˜}{\hat{z}}}_{k+1}$, i.o. the innovation factor, resulting in the a posteriori estimate ${\underset{˜}{\hat{x}}}_{\mathrm{aug},k+1}^{a}$. As a result, the analysis step can be expressed as follows:

(A17)
$${\underset{˜}{\chi }}_{i,k+1}^{z}=\underset{˜}{h}\left({\underset{˜}{\chi }}_{i,k+1}^{p}\right),\;1\leq i\leq n_{p}+1,$$

(A18)
$${\underset{˜}{\hat{z}}}_{k+1}=\sum_{i=1}^{n_{p}+1}\varepsilon {\underset{˜}{\chi }}_{i,k+1}^{z},$$

(A19)
$$B_{k+1}=\chi_{k+1}^{z}D_{\varepsilon }S^{T},$$

(A20)
$$U_{k+1}=P_{\varepsilon }+B_{k+1}R^{-1}{\left(B_{k+1}\right)}^{T},$$

(A21)
$$P_{k+1}^{a}=L_{k+1}U_{k+1}^{-1}{\left(L_{k+1}\right)}^{T},$$

(A22)
$${\underset{˜}{\hat{x}}}_{\mathrm{aug},k+1}^{a}={\underset{˜}{\hat{x}}}_{\mathrm{aug},k+1}^{p}+L_{k+1}U_{k+1}^{-1}B_{k+1}R^{-1}\left({\underset{˜}{y}}_{k+1}-{\underset{˜}{\hat{z}}}_{k+1}\right).$$

## Appendix C

**TABLE C1:** Results of the stability analysis performed by evaluating the Lyapunov function, which was determined using Krasovskii's method.

$c_{\mathrm{LV}}$ (‐)	0	0.1	0.2	0.3	0.4	0.5	0.6	0.7	0.8	0.9	1.0	1.1	1.2	1.3
*Stable?*	No	Yes	Yes	Yes	Yes	Yes	Yes	Yes	Yes	Yes	Yes	Yes	Yes	Yes
$c_{\mathrm{LV}}$ (‐)	1.4	1.5	1.6	1.7	1.8	1.9	2.0	2.1						
*Stable?*	Yes	Yes	Yes	Yes	Yes	Yes	Yes	No						
