# Supplementary material for: A Real‐Time Digital Twin for Human Cardiovascular Applications
Source: Int J Numer Method Biomed Eng. 2026 Sep 10;42(9):e70209. doi: 10.1002/cnm.70209 (PMC13561668; doi:10.1002/cnm.70209)
Supplement: Supplementary file 1 [file CNM-42-e70209-s001.docx]

Supplementary Material S1

The *in vivo* dataset consisted of 25 measurement series. For each measurement series, the estimated

$c_{LV}$, the LV$\frac{dp}{dt}$*|*_max_ and the administered dobutamine dose were plotted against each other. Both

the estimated $c_{LV}$ and the LV$\frac{dp}{dt}$*|*_max_ are depicted in two forms. First, a dotted representation that displays a parameter value for each 10 s time window, as explained in Section 2.3 and 2.6. Second, a representation with dashed lines showing a smoothed version of both parameters. This smoothed version was obtained using a third-order Savitzky-Golay filter with a window width of 25 samples. Lastly, the smoothed signals were used to calculate the Pearson correlation coefficient (*r*) between the estimated $c_{LV}$ and the LV$\frac{dp}{dt}$*|*_max_, which is reported in the lower-right corner of each plot.

**Figure S1:** Estimated $c_{LV}$, estimated $c_{LV}$(smoothed), LV$\frac{dp}{dt}$*|*_max_, LV$\frac{dp}{dt}|$_max_ (smoothed), and stepwise administered dobutamine depicted over time in patient 1 before TAVI (pre-TAVI). The Pearson correlation coefficient between estimated $c_{LV}$ (smoothed) and LV$\frac{dp}{dt}$*|*_max_ (smoothed) was *r* = 0*.*94, classified as strongly correlated (*r ≥* 0.7).

**Figure S2:** Estimated $c_{LV}$, estimated $c_{LV}$(smoothed), LV$\frac{dp}{dt}$*|*_max_, LV$\frac{dp}{dt}|$_max_ (smoothed), and stepwise administered dobutamine depicted over time in patient 1 after TAVI (post-TAVI). The Pearson correlation coefficient between estimated $c_{LV}$ (smoothed) and LV$\frac{dp}{dt}$*|*_max_ (smoothed) was *r* = 0*.*98, classified as strongly correlated (*r ≥* 0.7).

*dt*

**Figure S3:** Estimated $c_{LV}$, estimated $c_{LV}$(smoothed), LV$\frac{dp}{dt}$*|*_max_, LV$\frac{dp}{dt}|$_max_ (smoothed), and stepwise administered dobutamine depicted over time in patient 2 before TAVI (pre-TAVI). The Pearson correlation coefficient between estimated $c_{LV}$ (smoothed) and LV$\frac{dp}{dt}$*|*_max_ (smoothed) was *r* = 0*.*81, classified as strongly correlated (*r ≥* 0.7).

**Figure S4:** Estimated $c_{LV}$, estimated $c_{LV}$(smoothed), LV$\frac{dp}{dt}$*|*_max_, LV$\frac{dp}{dt}|$_max_ (smoothed), and stepwise administered dobutamine depicted over time in patient 2 after TAVI (post-TAVI). The Pearson correlation coefficient between estimated $c_{LV}$ (smoothed) and LV$\frac{dp}{dt}$*|*_max_ (smoothed) was *r* = 0*.*90, classified as strongly correlated (*r ≥* 0.7).

*dt*

**Figure S5:** Estimated $c_{LV}$, estimated $c_{LV}$(smoothed), LV$\frac{dp}{dt}$*|*_max_, LV$\frac{dp}{dt}|$_max_ (smoothed), and stepwise administered dobutamine depicted over time in patient 3 before TAVI (pre-TAVI). The Pearson correlation coefficient between estimated $c_{LV}$ (smoothed) and LV$\frac{dp}{dt}$*|*_max_ (smoothed) was *r* = *−*0*.*66, classified as moderate-to-weakly correlated (*r <* 0.7).

**Figure S6:** Estimated $c_{LV}$, estimated $c_{LV}$(smoothed), LV$\frac{dp}{dt}$*|*_max_, LV$\frac{dp}{dt}|$_max_ (smoothed), and stepwise administered dobutamine depicted over time in patient 3 after TAVI (post-TAVI). The Pearson correlation coefficient between estimated $c_{LV}$ (smoothed) and LV$\frac{dp}{dt}$*|*_max_ (smoothed) was *r* = 0*.*74, classified as strongly correlated (*r ≥* 0.7).

**Figure S7:** Estimated $c_{LV}$, estimated $c_{LV}$(smoothed), LV$\frac{dp}{dt}$*|*_max_, LV$\frac{dp}{dt}|$_max_ (smoothed), and stepwise administered dobutamine depicted over time in patient 4 before TAVI (pre-TAVI). The Pearson correlation coefficient between estimated $c_{LV}$ (smoothed) and LV$\frac{dp}{dt}$*|*_max_ (smoothed) was *r* = 0*.*96, classified as strongly correlated (*r ≥* 0.7).

**Figure S8:** Estimated $c_{LV}$, estimated $c_{LV}$(smoothed), LV$\frac{dp}{dt}$*|*_max_, LV$\frac{dp}{dt}|$_max_ (smoothed), and stepwise administered dobutamine depicted over time in patient 4 after TAVI (post-TAVI). The Pearson correlation coefficient between estimated $c_{LV}$ (smoothed) and LV$\frac{dp}{dt}$*|*_max_ (smoothed) was *r* = 0*.*27, classified as moderate-to-weakly correlated (*r <* 0.7).

**Figure S9:** Estimated $c_{LV}$, estimated $c_{LV}$(smoothed), LV$\frac{dp}{dt}$*|*_max_, LV$\frac{dp}{dt}|$_max_ (smoothed), and stepwise administered dobutamine depicted over time in patient 5 before TAVI (pre-TAVI). The Pearson correlation coefficient between estimated $c_{LV}$ (smoothed) and LV$\frac{dp}{dt}$*|*_max_ (smoothed) was *r* = 0*.*70, classified as strongly correlated (*r ≥* 0.7).

**Figure S10:** Estimated $c_{LV}$, estimated $c_{LV}$(smoothed), LV$\frac{dp}{dt}$*|*_max_, LV$\frac{dp}{dt}|$_max_ (smoothed), and stepwise administered dobutamine depicted over time in patient 5 after TAVI (post-TAVI). The Pearson

correlation coefficient between estimated $c_{LV}$ (smoothed) and LV$\frac{dp}{dt}$*|*_max_ (smoothed) was *r* = *−*0*.*56,

classified as moderate-to-weakly correlated (*r <* 0.7).

**Figure S11:** Estimated $c_{LV}$, estimated $c_{LV}$(smoothed), LV$\frac{dp}{dt}$*|*_max_, LV$\frac{dp}{dt}|$_max_ (smoothed), and stepwise administered dobutamine depicted over time in patient 6 before TAVI (pre-TAVI). The Pearson correlation coefficient between estimated $c_{LV}$ (smoothed) and LV$\frac{dp}{dt}$*|*_max_ (smoothed) was *r* = 0*.*55, classified as moderate-to-weakly correlated (*r <* 0.7).

**Figure S12:** Estimated $c_{LV}$, estimated $c_{LV}$(smoothed), LV$\frac{dp}{dt}$*|*_max_, LV$\frac{dp}{dt}|$_max_ (smoothed), and stepwise administered dobutamine depicted over time in patient 6 after TAVI (post-TAVI). The Pearson

correlation coefficient between estimated $c_{LV}$ (smoothed) and LV$\frac{dp}{dt}$*|*_max_ (smoothed) was *r* = 0*.*51,

classified as moderate-to-weakly correlated (*r <* 0.7).

**Figure S13:** Estimated $c_{LV}$, estimated $c_{LV}$(smoothed), LV$\frac{dp}{dt}$*|*_max_, LV$\frac{dp}{dt}|$_max_ (smoothed), and stepwise administered dobutamine depicted over time in patient 7 before TAVI (pre-TAVI). The Pearson correlation coefficient between estimated $c_{LV}$ (smoothed) and LV$\frac{dp}{dt}$*|*_max_ (smoothed) was *r* = 0*.*79, classified as strongly correlated (*r ≥* 0.7).

**Figure S14:** Estimated $c_{LV}$, estimated $c_{LV}$(smoothed), LV$\frac{dp}{dt}$*|*_max_, LV$\frac{dp}{dt}|$_max_ (smoothed), and stepwise administered dobutamine depicted over time in patient 7 after TAVI (post-TAVI). The Pearson

correlation coefficient between estimated $c_{LV}$ (smoothed) and LV$\frac{dp}{dt}$*|*_max_ (smoothed) was *r* = 0*.*92,

classified as strongly correlated (*r ≥* 0.7).

**Figure S15:** Estimated $c_{LV}$, estimated $c_{LV}$(smoothed), LV$\frac{dp}{dt}$*|*_max_, LV$\frac{dp}{dt}|$_max_ (smoothed), and stepwise administered dobutamine depicted over time in patient 8 before TAVI (pre-TAVI). The Pearson correlation coefficient between estimated $c_{LV}$ (smoothed) and LV$\frac{dp}{dt}$*|*_max_ (smoothed) was *r* = 0*.*84, classified as strongly correlated (*r ≥* 0.7).

**Figure S16:** Estimated $c_{LV}$, estimated $c_{LV}$(smoothed), LV$\frac{dp}{dt}$*|*_max_, LV$\frac{dp}{dt}|$_max_ (smoothed), and stepwise administered dobutamine depicted over time in patient 8 after TAVI (post-TAVI). The Pearson

correlation coefficient between estimated $c_{LV}$ (smoothed) and LV$\frac{dp}{dt}$*|*_max_ (smoothed) was *r* = 0*.*50,

classified as moderate-to-weakly correlated (*r <* 0.7).

**Figure S17:** Estimated $c_{LV}$, estimated $c_{LV}$(smoothed), LV$\frac{dp}{dt}$*|*_max_, LV$\frac{dp}{dt}|$_max_ (smoothed), and stepwise administered dobutamine depicted over time in patient 9 before TAVI (pre-TAVI). The Pearson correlation coefficient between estimated $c_{LV}$ (smoothed) and LV$\frac{dp}{dt}$*|*_max_ (smoothed) was *r* = 0*.*73, classified as strongly correlated (*r ≥* 0.7).

**Figure S18:** Estimated $c_{LV}$, estimated $c_{LV}$(smoothed), LV$\frac{dp}{dt}$*|*_max_, LV$\frac{dp}{dt}|$_max_ (smoothed), and stepwise administered dobutamine depicted over time in patient 9 after TAVI (post-TAVI). The Pearson

correlation coefficient between estimated $c_{LV}$ (smoothed) and LV$\frac{dp}{dt}$*|*_max_ (smoothed) was *r* = 0*.*83,

classified as strongly correlated (*r ≥* 0.7).

**Figure S19:** Estimated $c_{LV}$, estimated $c_{LV}$(smoothed), LV$\frac{dp}{dt}$*|*_max_, LV$\frac{dp}{dt}|$_max_ (smoothed), and stepwise administered dobutamine depicted over time in patient 10 before TAVI (pre-TAVI). The

Pearson correlation coefficient between estimated $c_{LV}$ (smoothed) and LV$\frac{dp}{dt}$*|*_max_ (smoothed) was

*r* = 0*.*88, classified as strongly correlated (*r ≥* 0.7).

**Figure S20:** Estimated $c_{LV}$, estimated $c_{LV}$(smoothed), LV$\frac{dp}{dt}$*|*_max_, LV$\frac{dp}{dt}|$_max_ (smoothed), and stepwise administered dobutamine depicted over time in patient 11 before TAVI (pre-TAVI). The

Pearson correlation coefficient between estimated $c_{LV}$ (smoothed) and LV$\frac{dp}{dt}$*|*_max_ (smoothed) was

*r* = 0*.*90, classified as strongly correlated (*r ≥* 0.7).

**Figure S21:** Estimated $c_{LV}$, estimated $c_{LV}$(smoothed), LV$\frac{dp}{dt}$*|*_max_, LV$\frac{dp}{dt}|$_max_ (smoothed), and stepwise administered dobutamine depicted over time in patient 11 after TAVI (post-TAVI). The

Pearson correlation coefficient between estimated $c_{LV}$ (smoothed) and LV$\frac{dp}{dt}$*|*_max_ (smoothed) was

*r* = 0*.*38, classified as moderate-to-weakly correlated (*r <* 0.7).

**Figure S22:** Estimated $c_{LV}$, estimated $c_{LV}$(smoothed), LV$\frac{dp}{dt}$*|*_max_, LV$\frac{dp}{dt}|$_max_ (smoothed), and stepwise administered dobutamine depicted over time in patient 13 before TAVI (pre-TAVI). The

Pearson correlation coefficient between estimated $c_{LV}$ (smoothed) and LV$\frac{dp}{dt}$*|*_max_ (smoothed) was

*r* = 0*.*72, classified as strongly correlated (*r ≥* 0.7).

**Figure S23:** Estimated $c_{LV}$, estimated $c_{LV}$(smoothed), LV$\frac{dp}{dt}$*|*_max_, LV$\frac{dp}{dt}|$_max_ (smoothed), and stepwise administered dobutamine depicted over time in patient 13 after TAVI (post-TAVI). The

Pearson correlation coefficient between estimated $c_{LV}$ (smoothed) and LV$\frac{dp}{dt}$*|*_max_ (smoothed) was

*r* = *−*0*.*22, classified as moderate-to-weakly correlated (*r <* 0.7).

**Figure S24:** Estimated $c_{LV}$, estimated $c_{LV}$(smoothed), LV$\frac{dp}{dt}$*|*_max_, LV$\frac{dp}{dt}|$_max_ (smoothed), and stepwise administered dobutamine depicted over time in patient 14 before TAVI (pre-TAVI). The

Pearson correlation coefficient between estimated $c_{LV}$ (smoothed) and LV$\frac{dp}{dt}$*|*_max_ (smoothed) was

*r* = 0*.*93, classified as strongly correlated (*r ≥* 0.7).

**Figure S25:** Estimated $c_{LV}$, estimated $c_{LV}$(smoothed), LV$\frac{dp}{dt}$*|*_max_, LV$\frac{dp}{dt}|$_max_ (smoothed), and stepwise administered dobutamine depicted over time in patient 14 after TAVI (post-TAVI). The

Pearson correlation coefficient between estimated $c_{LV}$ (smoothed) and LV$\frac{dp}{dt}$*|*_max_ (smoothed) was

*r* = 0*.*55, classified as moderate-to-weakly correlated (*r <* 0.7).
